# A Radio-Map Automatic Construction Algorithm Based on Crowdsourcing

**DOI:** 10.3390/s16040504

**Published:** 2016-04-09

**Authors:** Ning Yu, Chenxian Xiao, Yinfeng Wu, Renjian Feng

**Affiliations:** Key Laboratory of Education Ministry for Precision Opto-Mechatronics Technology, School of Instrumentation Science and Opto-Electronics Engineering, Beijing University of Aeronautics and Astronautics (Beihang University), Beijing 100191, China; xiaochenxian@buaa.edu.cn (C.X.); yfwu@buaa.edu.cn (Y.W.); rjfeng@buaa.edu.cn (R.F.)

**Keywords:** indoor localization, smartphone sensors, crowdsourcing, radio-map

## Abstract

Traditional radio-map-based localization methods need to sample a large number of location fingerprints offline, which requires huge amount of human and material resources. To solve the high sampling cost problem, an automatic radio-map construction algorithm based on crowdsourcing is proposed. The algorithm employs the crowd-sourced information provided by a large number of users when they are walking in the buildings as the source of location fingerprint data. Through the variation characteristics of users’ smartphone sensors, the indoor anchors (doors) are identified and their locations are regarded as reference positions of the whole radio-map. The AP-Cluster method is used to cluster the crowdsourced fingerprints to acquire the representative fingerprints. According to the reference positions and the similarity between fingerprints, the representative fingerprints are linked to their corresponding physical locations and the radio-map is generated. Experimental results demonstrate that the proposed algorithm reduces the cost of fingerprint sampling and radio-map construction and guarantees the localization accuracy. The proposed method does not require users’ explicit participation, which effectively solves the resource-consumption problem when a location fingerprint database is established.

## 1. Introduction

Wireless sensor networks (WSNs), which are multi-hop self-organized networks with wireless communication, are used to achieve information collection and processing by deploying a large number of tiny sensor nodes in a surveillance area. One of the fundamental functions in a wireless sensor network is to acquire location information of users, as the effectiveness of sensor monitoring depends mostly on whether the location information is accurate or not [1]. In the outdoor environment, the global positioning system (GPS) [2] is usually used to locate static or mobile users. As for the complex indoor environments, static or mobile users can be located by the received signal strength (RSS) [3], time of arrival (TOA) [4], time difference of arrival (TDOA) [5] and angle of arrival (AOA) [6] based methods.

In recent years, with the continuous development of wireless communication technology, WiFi is now deployed in many public places and has become a necessary infrastructure in people’s daily life [7,8]. Thus, WiFi-based indoor localization methods with location fingerprints [9,10,11,12,13] are commonly used for indoor localization. The WiFi information received by sensors at certain a indoor location is called a “fingerprint”, which is used to identify its corresponding location. The traditional fingerprint-based localization methods have two phases: an offline phase for acquiring a fingerprint database and an online phase for localization. In the offline phase, sampling points are set at regular distances in the indoor area, and the corresponding WiFi information, including media access control (MAC) addresses of access points (AP) and their corresponding RSS values at every sampling point is received and recorded. Each piece of Wifi information and its corresponding location coordinates constitute a location fingerprint, and all the location fingerprints constitute a radio-map of the indoor area. In the online phase, when a user sends a localization enquiry, the user’s current WiFi information is compared with the WiFi information in the radio-map. The location in the radio-map which WiFi information is most similar to the user’s current WiFi information is estimated to be the user’s current location.

The quantity of data in a radio-map directly impacts the indoor localization accuracy since the radio-map is a bridge connecting the offline phase and the online phase. During the offline phase, multiple measurement samples are required from a single sampling point in order to get a better localization accuracy. The manual data sampling method is resource-consuming and a practical bottleneck in radio-map construction. Every time a new localization demanding area is added, some new APs join in the network, or a certain AP moves to another new place [14], repetitive manual sampling work is needed to ensure the localization accuracy.

Generally, people carry out their daily work with smartphones in hand in indoor spaces. With the development of embedded technology and wireless communication technology, existing mobile phones are equipped with various sensors (like acceleration sensors, electronic compasses, gyroscopes, *etc.*) and good communication transceiver modules [15], which provide an important information interface connecting the wireless network with the actual environment, and reflect information about human activities in the environment to the wireless network. If all smart phones carried by ordinary users implicitly take part in the construction of a radio-map and contribute data to the acquisition of indoor fingerprints, the sampling workload can be significantly reduced. Therefore, in order to solve the poor extensibility and flexibility of radio-map construction in traditional fingerprint-based indoor localization algorithms, a novel automatic radio-map construction algorithm based on crowdsourcing (RACC) is proposed in this paper. The main contributions are summarized as follows.

(1) Data is collected by exploiting crowdsourcing technologies. The daily activities of users are represented by their walkable mobile phone path in the indoor building. The WiFi location information that a user has passed by, user’s walking acceleration, walking direction and rotational angular velocity are recorded by phones automatically. The recorded data provided by a large number of users are named crowdsourced data.
(2) Through the variation characteristics of users’ smartphone sensors, the indoor anchors (doors) are identified and their locations are regarded as reference positions of the radio-map. The crowdsourced data are clustered to acquire the representative fingerprints. According to the reference positions and the similarity between fingerprints, the representative fingerprints are linked to their corresponding physical locations and the radio-map is generated.
(3) Online localization is realized based on the constructed radio-map. Extensive simulations show that RACC can automatically generate radio-maps with acceptable accuracy and efficiently solve the resource-consuming problem in traditional radio-map-based indoor localization methods.

The remainder of the paper is organized as follows: Section 2 discusses two categories of methods in radio-map-based indoor localization algorithms. In Section 3, methodology and system architecture of the proposed algorithm is given. Section 4 presents the detailed procedures of RACC. Section 5 shows the experimental results and a performance evaluation of RACC. Finally, Section 6 concludes the paper.

## 2. Related Works

### 2.1. Offline Fingerprinting-Based Localization

RADAR [16] is an indoor localization system based on RSS fingerprints proposed by Microsoft Research in 2000. It is a deterministic localization method. In the offline phase, the mean value of received signal strength at every sampling point is recorded into the radio-map; in the online phase, the RSS information is matched to calculate the user’s current location.

Castro *et al.* [17] proposed a probabilistic location service for a WiFi networked environment called Nibble. Unlike RADAR, Nibble infers location information from the SNR measures received from the WiFi network, builds a probability distribution of signal space using Bayesian network and chooses the location with the maximum *a posteriori* probability to be the estimated location. The service can achieve a room-level localization accuracy. Roos *et al.* [18] considered two probabilistic approaches—kernel method and histogram method—to realize indoor localization. The RSS data at each reference point are recorded for a period of time and stored in the radio-map. The probability distribution characteristics of received signal strength can be respectively fitted by kernel function and histogram, and then user’s location is calculated. Compared with the deterministic localization method, probabilistic method has better noise immunity, and can largely reduce the effects of noise on the localization accuracy. Youssef *et al.* [19] designed the Horus location determination system. In the data collection phase, the mean value and standard deviation of received signal strength at every reference point are recorded. The collected data are modeled by a Gaussian probability distribution and the locations that receive the same AP signals are grouped using the clustering method to improve the online search efficiency.

Brunato *et al.* [20] presented a statistical learning theory for mobile user location estimation. The offline sampling data are set as input vectors, the corresponding coordinates are output vectors, and thus the linear decision function is constructed by support vector machine (SVM) in high dimensional space. The user’s location can be obtained by inputting the user’s RSS information to the linear function. Battiti *et al.* [21] considered a multi-layer perceptron (MLP) architecture for building a flexible mapping between the raw signal measurements collected offline and the locations of the sampling points. Laoudias *et al.* [22] proposed an indoor localization algorithm based on clustered RBF (cRBF). It applied a clustering technique into the reference fingerprints, reduced dramatically the number of neurons in the hidden layer and then improved the training performance of the offline fingerprinting data.

Yin *et al.* [23] proposed a novel method to adapt the temporal radio-maps for indoor location estimation by offsetting the varying environmental factors. It continuously adjusts the fingerprint information of reference points through a regression analysis, providing more accurate data references for online localization. Pan *et al.* [24] developed a graph-based semi-supervised learning method to learn mobile users’ locations. A manifold-based model is firstly built from the data recorded in the offline training phase and then in the online localization phase the weighted k-nearest neighbor method is used to localize mobile clients. Au *et al.* [25] proposed an indoor tracking system based on compressive sensing (CS). The system clusters the RSS fingerprints and selects fingerprint representatives after building the radio-map offline. The online phase is a two-step method, where first a coarse localization step is performed to estimate the approximate location of the user by matching user’s RSS information to the fingerprint representatives, and then the accurate location is estimated in the fine localization step formulating the localization problem into a sparse-natured signal recovery problem, for which the CS theory can be applied. Liu *et al.* [26] proposed a localization solution based on visible light communication. It employed LED lights as beacons and constructed Bayesian framework through Gaussian process to realize the low-cost localization.

All of the methods mentioned above require manpower to do the offline sampling work. The differences are that they employ different matching methods to realize the online localization on the basis of generated radio-map.

### 2.2. Offline Fingerprinting-Free Localization

Considering the problem that manual offline fingerprint acquisition is resource-consuming and easily affected by environmental changes, scholars have put forward many alternatives to realize radio-map construction.

Based on users’ point of view, some researchers allow users to join in the process of radio-map construction directly. Bolliger *et al.* [27] proposed an adaptive zero-configuration indoor localization system through user collaboration. The style that Redpin uses is similar to the Wikipedia concept for information collection, that is, a single locally-knowledgeable user is allowed to associate any text string with a place’s fingerprint and many visitors can then rely on the database of fingerprints. Lee *et al.* [28] developed a crowdsourcing approach for indoor place recognition. An open participatory system through which general users can contribute fingerprints is built and the place recognition service can be provided as the database expands to certain degree. To deal with the volatility of RSS data and the radio-map redundancy, Yang *et al.* [29] proposed a localization method FreeLoc, that maintained only one fingerprint for a location in a radio-map, regardless of any number of uploaded data sets for a given location. It provides consistent localization accuracy in an environment where there exist device heterogeneity and the multiple surveyor problems. Park *et al.* [30] grew an organic indoor location system called OIL, which explicitly incorporated site surveys by users. The organic localization start with an empty database which is gradually populated with user-provided fingerprints. On the basis of OIL, Ledlie *et al.* [31] described a scalable user-generated localization engine named Molé. It arranges places uploaded by a number of users in a hierarchy and utilizes the Maximum Overlap (MAO) to estimate the place as the one whose fingerprint is most similar to the user’s fingerprint. Sorour *et al.* [32] gave a reduced-effort radio-map generation algorithm based on dimensionality reduction transfer learning. The location of unlabeled crowdsourced signal observations can be estimated through manifold alignment and thus the indoor radio-map is constructed, which significantly reduces the large calibration load.

Other researchers think that user motions can be applied to connect previously independent RSS fingerprints since the increasing number of sensors on mobile phones offer new opportunities for indoor localization. Azizyan *et al.* [33] proposed SurroundSense, which is a mobile phone-based system that explores localization via ambience fingerprinting. It assumed that the combined effect of ambient sound, light and color can be unique enough for locating certain place. SVMs and color clustering are used to extract simple features from the collected data and then the fingerprint database is created. Rai *et al.* [14] developed a zero-effort indoor localization system called Zee. It leverages the sensors in smartphones carried by users to track them as they are moving in the indoor building and the user’s steps and orientations can be estimated. Armed with a map of the indoor space of interest, Zee employs WiFi and sensor measurements crowdsourced from users to construct a WiFi training set and then uses augmented particle filtering to simultaneously infer the user’s location. Wu *et al.* [34] designed WILL, an indoor localization method based on off-the-shelf WiFi infrastructure and mobile phones. WILL partitions the crowdsourced fingerprints into different virtual rooms and a logical floor plan is accordingly constructed. Finding a matching between logical and ground-truth floor plan is a key step for localization. Liu *et al.* [35] presented an indoor localization approach named LiFS, in which the calibration of fingerprints is crowdsourcd. LiFS leverages the user motions to link multiple separated RSS fingerprints and builds the mapping relation between the fingerprints space and the stress-free floor plan through multidimensional scaling (MDS), which leads to the automatic establishment of fingerprint database.

The indoor localization approaches mentioned in this section take advantage of auxiliary information such as user collaboration, sensor measurements and actual indoor maps to annotate RSS fingerprints with their corresponding location labels, and thus generate the radio-map without any offline manual fingerprinting work. However, the accuracy of radio-maps automatically generated by existing indoor localization systems still needs to be improved when it is compared with the accuracy of manually constructed ones. In this paper, a novel radio-map automatic construction algorithm based on crowdsourcing (RACC) is proposed. For the promise that the localization accuracy is acceptable, it simply employs the WiFi and sensor data crowdsourced by users’ smartphones, to construct the radio-map automatically without explicit volunteers to bind RSS fingerprints to a location.

## 3. Methodology & System Architecture

**Figure 1:**
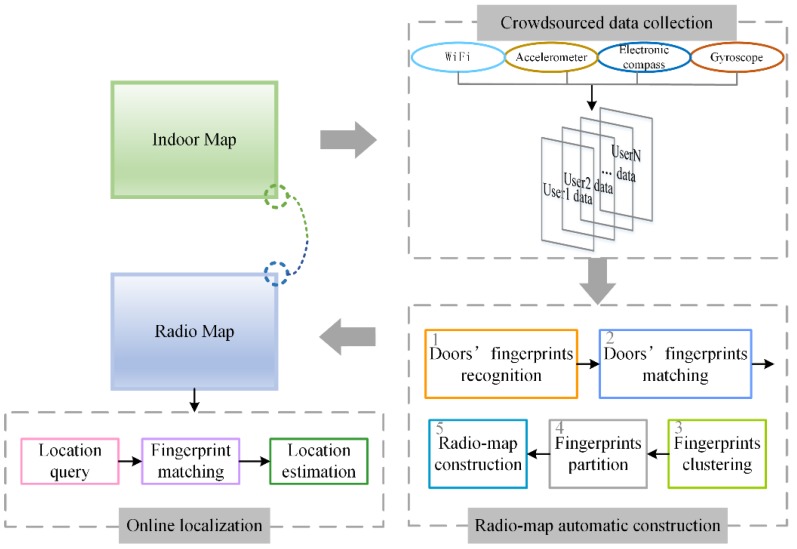
Schematic diagram of proposed algorithm RACC.

The proposed algorithm RACC consists of three parts: crowdsourced data collection, radio-map automatic construction and online localization, as is shown in Figure 1. The emphasis is on radio-map automatic construction.

The crowdsourced data include WiFi information (also called RSS fingerprints), user’s walking acceleration, walking direction and rotational angular velocity. The radio-map automatic construction part is divided into five sections: doors’ fingerprint recognition, door’s fingerprint matching, crowdsourced fingerprints clustering, fingerprints partition and radio-map construction. The online localization part covers three sections: location enquiry, fingerprint matching and location estimation.

### 3.1. Crowdsourced Data Collection

Unlike the traditional manual sampling way, RACC gathers general users’ contributions to obtain raw fingerprints, namely using crowdsourcing. When users walk in the indoor space for their daily activities, the smartphones carried by them can collect the WiFi features of the locations that users have passed. Simultaneously, a suite of sensor measurements like user’s walking acceleration, walking direction and rotational angular velocity, which reflect users’ walking characteristics, are also recorded by the sensors (acceleration sensor, electronic compass, gyroscope, *etc.*) in smartphones. The collection of the RSS fingerprints and sensor data is performed automatically in the background of phones and is transparent to the users, but has no impact on the users’ daily activities. The crowdsourcing way makes full use of the crowd’s effort and therefore successfully accomplishes the cost reduction in manpower.

**Figure 2:**
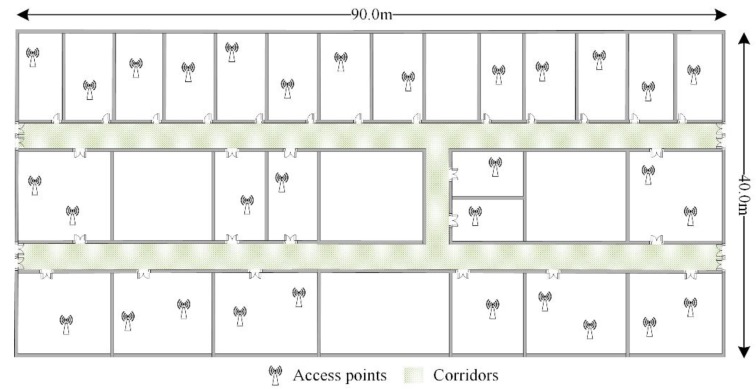
Floor plan of experimental areas.

**Figure 3:**
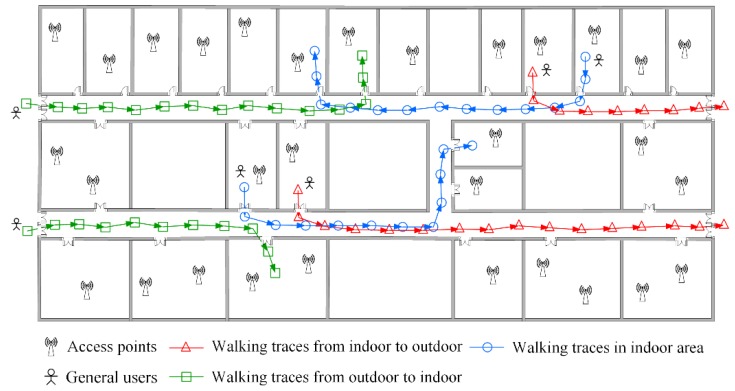
Users’ moving pattern in indoor areas.

The floor plan where the data are crowdsourced is shown in Figure 2. In the indoor area, there are several APs deployed in the rooms and their locations are unknown. The patterns that users walk in or out the indoor area, or traverse in the indoor area are displayed in Figure 3. The triangle, square and circle in Figure 3 all represent the locations where the crowdsourced data are recorded during the period users are moving. Since the GPS signal would be lost or appear temporarily once users set foot inside or outside the indoor area [36], the obvious change can be used to pick out the data collected in the indoor area. Meanwhile, their fingerprints in different building entrances are acquired and recorded in advance to make a distinction among them for the later work.

The smartphones would record the RSS fingerprints, user’s walking acceleration, walking direction and rotational angular velocity at fixed sampling period (e.g., every one second) automatically in the background, which are formatted in Table 1.

(1) User ID. Every user has an unique ID, which is utilized to identify the crowdsourced data provided by different users.
(2) RSS fingerprint. RSS fingerprint includes the MAC addresses of APs {*ap_1_*,*ap_2_*,…}_*ti*_ and their corresponding RSS values{*rss_i1_*,*rss_i2_*,…}.
(3) Walking acceleration. User’s walking acceleration *acc* = {*acc_X_*,*acc_Y_*,*acc_Z_*} is measured by an acceleration sensor, where *acc_X_*, *acc_Y_* and *acc_Z_* are respectively the acceleration components in the X, Y and Z direction.
(4) Walking direction. The embedded electronic compass of smartphone measures three components: the angle relative to the magnetic north, the angle between x-axis orientation of smartphones and the horizontal plane, the angle between y-axis orientation of smartphones and the horizontal plane. On account of different placement of smartphones when users are moving, only the angle relative to the magnetic north is recorded into the crowdsourced data.
(5) Rotational angular velocity. Users’ rotational angular velocity *rot* = {*rot_X_*,*rot_Y_*,*rot_Z_*} is measured by the gyroscope in smartphones. The rotational angular velocity in X, Y and Z orientation would all get a change when users make a turn due to the different placement of smartphones, but one of the three parameters changes more evidently than the other two.

### 3.2. Automatic Construction of Radio Map

The goal of RACC is to extract several representative RSS fingerprints from the crowdsourced RSS fingerprints and to annotate them with physical locations according to some rules, then automatically construct a radio-map of the indoor space. In general, users are more concerned about the paths to their destinations rather than the activity locations which users have already reached. For example, customers care more for how to reach places (such as clothing stores, coffee shops or washrooms) from the building entrances or their current positions in the shopping mall. They usually don't need positioning guide after getting to the destination where their activities are more random and subjective based on their interests. Therefore our algorithm aims to generate a radio-map of indoor public walking paths (like corridors), that is, the fingerprints in a radio-map merely cover the corridor area. When users arrive at rooms (guided by the constructed radio map) after walking pass corridors from the entrance or certain location in corridor, the location guidance in rooms is no longer carried out.

The automatic construction of radio-maps goes through five steps: recognizing doors’ fingerprints, labeling doors’ fingerprints, clustering crowdsourced fingerprints, partitioning fingerprint representatives and constructing a radio-map. Some features which imply users’ physical locations exist in the crowdsourced data, for instance, the walking direction would make a big turn when users move through a door of the room, walking in or out the room. On this basis, the doors can be recognized and fingerprints near doors are recorded. Furthermore, since the positions of room doors hardly change, it can provide a fixed reference (anchors) for the automatic matching of other fingerprints. The locations that users walk in daily life almost cover all the indoor paths (corridors), therefore, a huge volume of crowdsourced data are produced. The crowdsourced fingerprints is clustered in the clustering step based on the similarities among RSS fingerprints and the representative fingerprints are selected. On the basis of the fixed reference (doors’ locations and their corresponding fingerprints) and the similarty between fingerprints, the representative fingerprints can be matched to the corresponding physical locations in the area, and then the radio-map of the whole area is constructed.

### 3.3. Online Localization

When users query their current positions, users’ current RSS fingerprints will be searched and matched with RSS fingerprints in the radio-map to find the most similar (nearest) fingerprints, and then the location information of the most similar (nearest) fingerprints are sent back to the user to finalize the localization. In RACC, the weighted k-nearest neighbor method is utilized to realize the online localization. When the user sends a location query *RSS_query_*, similar fingerprints in the radio-map will be searched. Here given that there is a fingerprint chain with *k* similar RSS fingerprints that are considered to be similar with *RSS_query_*, that is, {*RSS_1_*,*RSS_2_*,…,*RSS_k_*}. The weight of each RSS fingerprint in the chain is assigned according to its similarity with *RSS_query_*. The more similar they are, the larger the weight is. The weight can be calculated as:

(1) $$\omega_{i}=\frac{\frac{1}{{\left\|RSS_{i}-RSS_{query}\right\|}_{2}+\varepsilon }}{\sum_{i=1}^{k}\frac{1}{{\left\|RSS_{i}-RSS_{query}\right\|}_{2}+\varepsilon }}$$

where *ε* is a near-zero positive. It is used to make the calculation effectively when Euclidean distance between RSS fingerprints ‖*RSS_i_*-*RSS_query_*‖_2_ = 0. The geographical location of *RSS_query_* can be obtained as:

(2) $$C_{\text{query}}=\sum_{i=1}^{k}\omega_{i}\cdot C_{i}$$

where *C_i_* (*i* = 1,2,…,*k*) is the corresponding geographical location of *k* similar fingerprints.

## 4. RACC Algorithm

### 4.1. Doors’ Fingerprint Recognition

**Figure 4:**
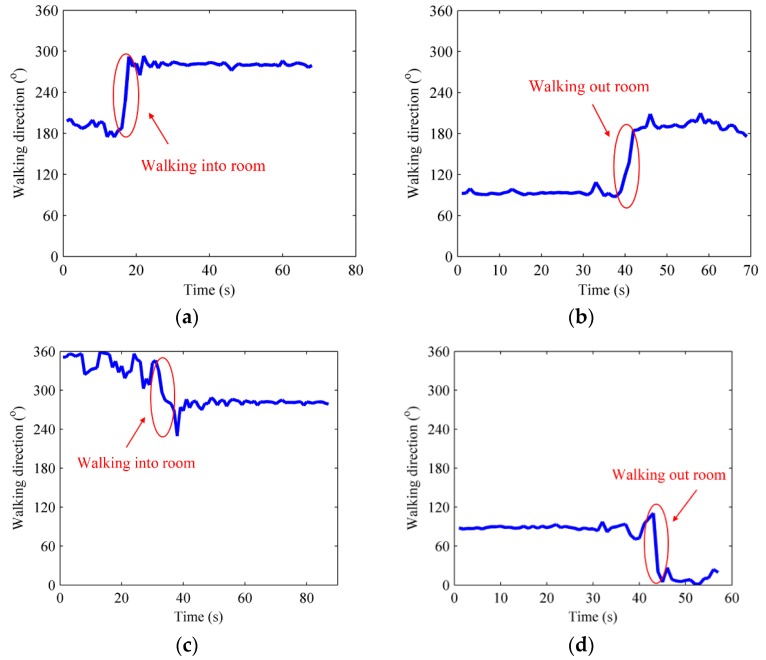
The variation tendencies of electronic compass. (**a**) Walk in room from the right; (**b**) Walk out of room from the right; (**c**) Walk in room from the left; (**d**) Walk out of room from the left.

**Figure 5:**
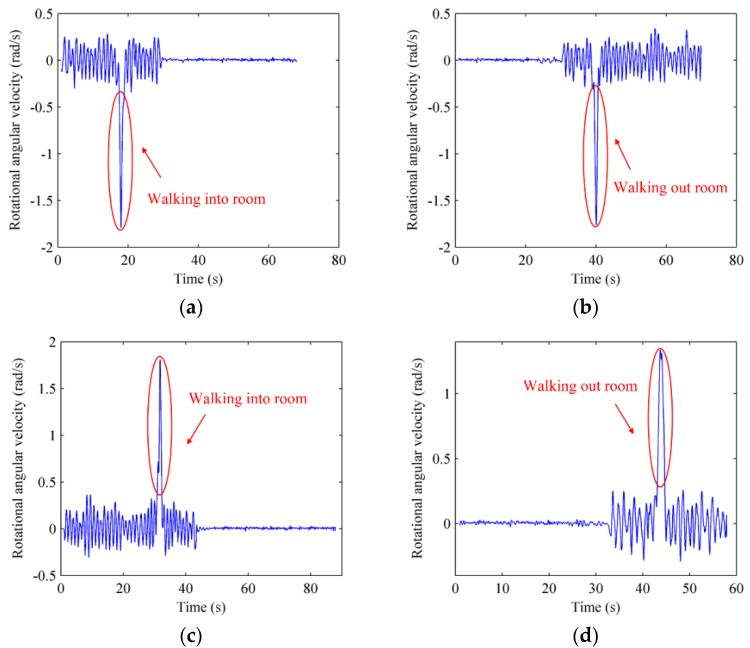
The variation tendencies of gyroscope. (**a**) Walk in room from the right; (**b**) Walk out of room from the right; (**c**) Walk in room from the left; (**d**) Walk out of room from the left.

Users’ walking direction and rotational angular velocity would change greatly when users traverse the door in or out of a room. The variation feature helps us to recognize when users are just at the position of certain door and what are the corresponding recorded RSS fingerprints. The statistic variation tendencies of electronic compass and gyroscope when users walk through doors in different directions are displayed in Figure 4 and Figure 5.

Figure 4a and Figure 5a demonstrate the changing electronic compass and gyroscope signals when users walk into a room from the right; Figure 4b and Figure 5b show the changing electronic compass and gyroscope signals when users walk out of the room from the right; Figure 4c and Figure 5c show the electronic compass and gyroscope changes when users walk into the room from the left; Figure 4d and Figure 5d show the changing electronic compass and gyroscope signals when users walk out of the room from the left. From Figure 4, it can be seen that the electronic compass measurements would fluctuate within a small range when users are moving straight, but they change dramatically when users are walking through doors. The change angle is about 70° to 110° when users walk through doors because of the vertical intersecting relationship between corridor and door. Figure 5 manifests the fact that the rotational angular velocity measured by a gyroscope also shows an suddern change when users walk through doors, and the change when users walk from the right is the opposite from that when users walk from the left, so the moments user walk in or out of rooms can be accurately recognized based on the changing features in crowdsourced electronic compass and gyroscope measurements, and the corresponding RSS fingerprints recorded at those moments can be obtained at the same time.

According to the variation characteristics discussed above, we make the following rules to realize the recognition of doors and extraction of their RSS fingerprints:

Rule 1: The difference of walking direction Δ*cp*_12_ and the difference of rotational angular velocity Δ*rot*_12_ between two adjacent moments *t*_1_ and *t*_2_ (*t*_2_ > *t*_1_) respectively are:

(3) $$\Delta {cp}_{12}={\left\|{cp}_{t_{2}}-{cp}_{t_{1}}\right\|}_{2}$$

(4) $$\Delta {rot}_{12}={\left\|{rot}_{t_{2}}-{rot}_{t_{1}}\right\|}_{2}$$

If Δ*cp*_12_ and Δ*rot*_12_ both satisfy the conditions Δ*cp*_12_ > *ε_cp_* and Δ*rot*_12_ > *ε_rot_*, *t*_2_ is considered to be the moment when users walk through doors, where *ε_cp_* and *ε_rot_* are the predefined thresholds which are determined by the change of walking direction and rotational angular velocity respectively. The RSS fingerprint at *t*_2_ is considered to be the candidate fingerprint of a certain room door.

Rule 2: For the two doors’ candidate fingerprints *RSS*_1_ = {*rss*_11_,*rss*_12_,…,*rss*_1*n*_} and *RSS*_2_ = {*rss*_21_,*rss*_22_,…,*rss*_2*n*_}, define the RSS similarity as:

(5) $$\Delta {RSS}_{12}={\left\|{RSS}_{1}-{RSS}_{2}\right\|}_{2}=\sqrt{\frac{1}{n}\sum_{i-1}^{n}{\left({rss}_{1i}-{rss}_{2i}\right)}^{2}}$$

Given that *ε_RSS_* is the predefined threshold. If Δ*RSS*_12_ < *ε_RSS_*, the candidate fingerprints *RSS*_1_ and *RSS*_2_ are similar and they are labeled with the same door. If Δ*RSS*_12_ > *ε_RSS_* , the candidate fingerprints *RSS*_1_ and *RSS*_2_ are dissimilar and they are labeled with different doors.

*ε_RSS_* represents the average maximum dissimilarity of RSS fingerprints in adjoining locations which is feasible to distinguish RSS fingerprints at different positions. The MAC addresses of APs and the corresponding RSS values recorded by two similar RSS fingerprints have few differences. Smartphones carried by multiple users would acquire similar RSS fingerprints when they walk through the same door. Following Rule 2, multiple similar fingerprints are assigned to the same room door. In order to facilitate the matching between door’s location with its corresponding fingerprints, the averaged value of multiple similar fingerprints are used to represent the door’s fingerprints, abbreviated as D-fingerprint. Rule 3 gives the calculation.

Rule 3: For *m* similar candidate fingerprints labeled with the same room door: *RSS*_1_ = {*rss*_11_,*rss*_12_,…,*rss*_1*n*_}, *RSS*_2_ = {*rss*_21_,*rss*_22_,…,*rss*_2*n*_}, …, *RSS_m_* = {*rss*_*m*1_,*rss*_*m*2_,…,*rss_mn_*}, define their averaged fingerprint as D-fingerprint:

(6) $$\begin{array}{l} RSS_{D}=\\ \{Avg\{rss_{11},rss_{21}\ldots ,rss_{i1},\ldots ,rss_{m1}\},Avg\{rss_{12},rss_{22}\ldots ,rss_{i2},\ldots ,rss_{m2}\},\ldots ,\\ Avg\{rss_{1j},rss_{2j}\ldots ,rss_{ij},\ldots ,rss_{mj}\},\ldots ,Avg\{rss_{1n},rss_{2n}\ldots ,rss_{in},\ldots ,rss_{mn}\}\} \end{array}$$

where *Avg*{·} is the average function.

The doors’ fingerprints can be extracted from the crowdsourced data based on Rules 1 and 2. However, there are two exceptions that need further explanation. One is that users could occasionally or deliberately make turns when they are walking straight, the other is that the electronic compass and gyroscope in smartphones would make the similar aforementioned change when users walk through the corridor intersections. The following describes these two cases.

If users make a turning action randomly someplace, it is difficult for the turning actions to appear in the same place, which means that the probability that the users make turning actions randomly at the same position is relatively low. Only when the number of users reached some degree and their walking direction and rotational angular velocity all change at the similar RSS fingerprints, it can be indicated that the users all have walked through a certain door. Thus, the occasional changing points of sensor measurements can be eliminated and have no influence on the identification of doors’ fingerprints.

**Figure 6:**
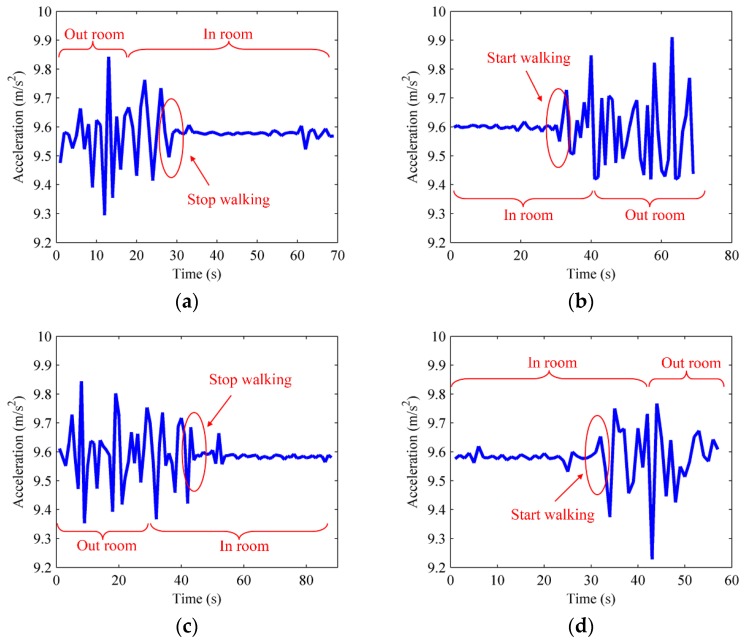
The variation tendencies of accelerometer. (**a**) Walk in room door on the right; (**b**) Walk out room door on the right; (**c**) Walk in room door on the left; (**d**) Walk out room door on the left.

As for the similar change of walking direction and rotational angular velocity when users walk through the corridor intersections, a method to differentiate doors from corridor intersections is given as follows: when users walk into rooms, they generally slow down or remain static due to the space constraint, and thus the acceleration tends to be gentle or stable. On the other hand, when users walk through corridor intersections, their acceleration remains unchanged on account of users’ continued walking. Therefore, the statistic changing tendencies in crowdsourced acceleration measurements can be used to discriminate the turning actions occurring at room doors from those occurred at corridor intersections. Figure 6 shows the changing rules of acceleration during the periods before and after users walk in or out of rooms.

From Figure 6, we can conclude that the acceleration is transformed from an obvious regular fluctuation to little fluctuation when users walk into rooms and become static. On the contrary, the acceleration changes from an initial steady state to a large range of fluctuation when users walk out of the rooms. Rule 4 is set to make the distinction of turnings between doors and corridor intersections.

Rule 4: Given *t* is the moment when walking direction and rotational angular velocity simultaneously make a great change. If the time period Δ*t*_static_ that users remain static or nearly static satisfies the condition Δ*t*_static_ > *T* after the moment *t*, then *t* is recognized as the moment when users walk into the rooms, where *T* is predefined time threshold. If the time period Δ*t*_static_ when users remain static or nearly static satisfies the condition Δ*t*_static_ > *T* before the moment *t*, then *t* is recognized as the moment when users walk out the rooms. Otherwise *t* is recognized as the moment when users go through corridor intersections.

One case that needs to be specified is that individual users might stop walking immediately after passing through the corridor intersections. This situation would not affect the judgement for Rule 4 because of the use of crowdsourced data. Since Rule 4 is concluded statistically from the same characteristics in a large amount of users’ walking paths, some unreasonable small probability trajectories of individual users can be removed effectively.

Based on the above rules, all doors’ fingerprints in the indoor area are extracted from the crowdsourced data. However, the doors’ fingerprints have not been annotated with their specific door number yet. It is necessary to match the D-fingerprints with their corresponding door number in order to get D-fingerprints’ physical locations.

### 4.2. Doors’ Fingerprint Matching

In practical conditions, users’ walking paths are constricted by the indoor architecture, and geographically there are distinct topology relationships between the doors. As is seen in Figure 3, the building entrances lie on the left side of floor plan. No matter which room users want to reach when they set foot in the indoor area from the entrances, they first have to go past the room door that is closer to the entrances and later go past the room door that is far from the entrances. The two neighboring doors are in a sequential constraint relationship in users’ walking path. As mentioned, different building entrances can be identified by the changes in GPS signal and the RSS fingerprints calibrated previously at entrances. Hence, we start fingerprint matching from the building entrances. In a single user’s walking path, the time and steps spent from the entrance to the door which is the closest to entrance are the minimum among those from the entrance to any other door. Taking advantage of this feature, the D-fingerprint of the nearest door to entrance is labeled first. In sequence, the D-fingerprint of the nearest door to the door which is successfully matched in the last matching round is labeled in the same way.

The movement acceleration will generate a peak with every step user takes and the users’ walking steps can be calculated by setting a threshold [35]. Rules 5 and 6 are drafted to fulfill the doors’ fingerprint matching in accordance with the principle that walking past two adjacent doors spends the minimum time and steps in a single user’s trajectory.

Rule 5: D-fingerprint’ matching of the nearest door to entrance. Given that *E* is one of the building entrances. The time {*t*_1_,*t*_2_,…,*t_s_*} and steps {*p*_1_,*p*_2_,…,*p_s_*} spent from *E* to each D-fingerprint in every users’ trajectory are computed. Taking the minimum of the time and steps as:

(7) $$t_{\text{min}}=\text{min}\{t_{1},t_{2},\ldots ,t_{s}\}$$

(8) $$p_{\text{min}}=\text{min}\{p_{1},p_{2},\ldots ,p_{s}\}$$

If *t*_min_ and *p*_min_ derived by all users correspond to the same D-fingerprint *RSS*_*D*1_, then *RSS*_*D*1_ is labeled with the door D_1_ which is closest to the entrance *E*.

Rule 6: Recursive D-fingerprint’ matching of adjacent doors. Given that the doors {*D*_1_,*D*_2_,…,*D_k_*} have already been matched with D-fingerprints. The time {*t*_1_,*t*_2_,…,*t_s_*} and steps {*p*_1_,*p*_2_,…,*p_s_*} spent from the door *D_k_* to any other D-fingerprint (not include the D-fingerprints {*rss*_1_,*rss*_2_,…,*rssk*} already labeled with the doors {*D*_1_,*D*_2_,…,*D_k_*}) in every users’ trajectory are computed. Taking the minimum of the time and steps:

(9) $$t_{\text{min}}=\text{min}\{t_{k+1},t_{k+2},\ldots ,t_{s}\}$$

(10) $$p_{\text{min}}=\text{min}\{p_{k+1},p_{k+2},\ldots ,p_{s}\}$$

**Figure 7:**
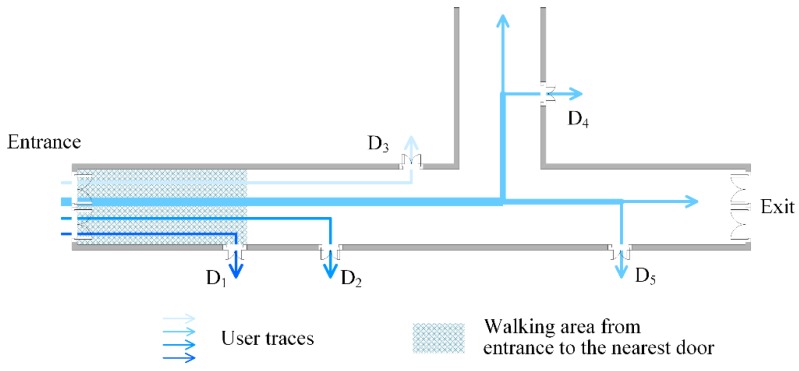
The D-fingerprint’s matching of the nearest door to the entrance.

**Figure 8:**
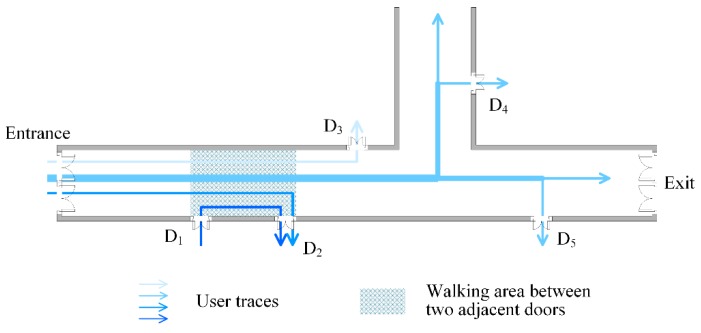
The recursive D-fingerprints’ matching of adjacent doors.

If *t*_min_ and *p*_min_ derived by all users correspond to the same D-fingerprint *RSS*_*Dk*+1_ , then *RSS*_*Dk*+1_ is labeled with the adjacent door *D*_*k*+1_ which is closest to the door *D_k_*. Figure 7 illustrates the D-fingerprint matching of the nearest door to entrance and Figure 8 illustrates the recursive D-fingerprint matching of adjacent doors.

(1) D-fingerprint’ matching of the nearest door to the entrance

In every user’s trajectory that passes through the entrance, the D-fingerprint which cost the least time and minimum steps is confirmed according to Rule 5. This D-fingerprint corresponds to the room door that is closest to the entrance and its physical position is the location of the door that is closest to the entrance, like door *D*_1_ shown in Figure 7.

(2) Recursive D-fingerprint’ matching of adjacent doors

In every user’s trajectory that passes through the last matched door, the next D-fingerprint which costs the least time and minimum steps to arrive is confirmed according to Rule 6. This D-fingerprint corresponds to the door that is closest to the last matched door D_1_ and its physical position is the location of the door that is closest to the last matched door D_1_, like the door D_2_ shown in Figure 8.

It should be noted that Rules 5 and 6 avoid precise computation of every users’ walking distance, and only the least time and minimum steps from the current D-fingerprint to the next D-fingerprint need to be found in order to match the door with the next D-fingerprints. Although different users spend different times and steps walking past two similar adjacent doors, the two doors are still the nearest neighbor in each individual user’s trajectory, accordingly, the time and steps of walking pass the two doors are the minimum. Based on crowdsourced data, two doors are confirmed to be the nearest neighbor if an overwhelming majority of users’ trajectories indicate so. Rule 5 and 6 exploit the local extremum method to bind D-fingerprints with their corresponding locations sequentially. Although Rules 5 and 6 do not necessarily ensure that *t*_min_ and *p*_min_ derived by all users should correspond to the same D-fingerprint, they are still workable when *t*_min_ and *p*_min_ of most users’ trajectories correspond to the same D-fingerprint based on the crowdsourcing principle.

**Figure 9:**
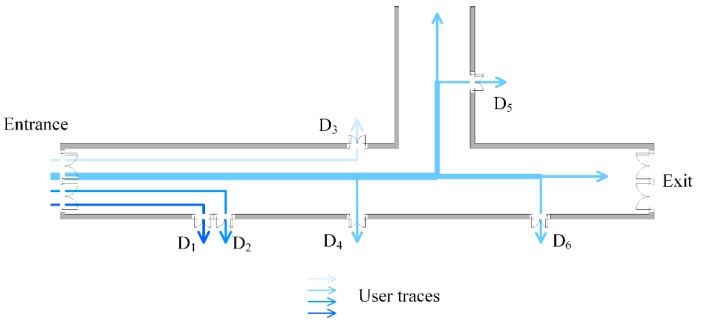
The D-fingerprints’ matching of doors at special positions.

As a building has multiple entrances, the adjacent topologies of all the D-fingerprints formed from different entrances are overlapped or complementary, which ensures the accuracy of doors’ fingerprint matching. Figure 9 shows two special cases of D-fingerprints’ matching. One case is that two doors are very close and the other case is that two doors are oppositely located. The two cases are analyzed respectively.

(1) For the case that D_1_ is close to D_2_, if D_1_ and D_2_ are closer than the coverage distance of a fingerprint (1~2 m), the fingerprints generated by D_1_ and D_2_ can be merged into one fingerprint, that is, they are calibrated as the same fingerprint. If D_1_ is far from D_2_, two different fingerprints will be generated separately, and the corresponding location of these two fingerprints will be identified according to their users’ sequential trajectories.
(2) For the case that D_3_ is opposite to D_4_, since walking direction and rotational angular velocity are different when users take turns in front of them, the fingerprints of D_3_ and D_4_ can be identified according to users’ trajectories.

These special location features, such as D_1_ and D_2_, or D_3_ and D_4_, provide more specific and deterministic references (anchors or landmarks) for the whole radio-map. The changes of the crowdsourced data in special locations are more characteristic, which is beneficial to the identification and matching of D-fingerprints.

### 4.3. Crowdsourced Fingerprint Clustering

Based on D-fingerprints and their physical locations identified in Section 4.2, the locations of other RSS fingerprints in a radio-map can be obtained through a certain method. However, since the crowdsourced RSS fingerprints are collected from a large number of users, and some users would walk through the same place at different times, it may happen in one case that more than one RSS fingerprint corresponds to the same or a neighboring physical location. The calculation cost to acquire all crowdsourced fingerprints’ geographical locations is huge, and moreover, the generated radio-map might be redundant and inaccurate, so this paper proposes to cluster the crowdsourced fingerprints by means of the similarity between RSS fingerprints, and pick out some RSS fingerprints to represent the RSS fingerprints in a neighboring small area. Considering the huge number of crowdsourced fingerprints, how many clusters that crowdsourced fingerprints are clustered into and how to select the fingerprint representatives optimally are the key points. Here the Affinity Propagation Cluster (AP-Cluster) method [37] is used to solve these problems. Compared with other cluster methods, AP-Cluster does not need the assignment of the number of clusters, which is determined globally by the similarities among data. The cluster representatives are selected from the original data and can adequately represent their subordinate data to some degree. The properties of AP-Cluster method meet our requirements of crowdsourced fingerprints clustering. AP-Cluster method assumes that all the data points form a network in which each data point can evaluate the others and deliver evaluation parameters. Every data point is considered to be the cluster representative in the initialization phase. In the process of clustering, every data point has a parameter to evaluate its support degree, and simultaneously assess whether it is suitable to be the representative combining with the support degree evaluation given by other data points. The data points who get the maximum support and suitability evaluation are chosen to be the cluster representatives.

According to the AP-Cluster method, all RSS fingerprints are assumed to be a network and each RSS fingerprint calculates the support and suitability evaluation of any fingerprint serving as a representative. The AP-Cluster method has two inputs. The similarity between a pairs of fingerprint *s*(*RSS_i_*,*RSS_k_*) = −Δ*RSS_ik_* (Δ*RSS_ik_* see Rule 2 in Section 4.1), also called clustering similarity, is one of the input parameters. The preference degree of data points being cluster representative *P_r_* (*P_r_* < 0) is another input parameter, which influences the number of clusters. The suitability evaluations of fingerprints are initialized to zero: *a*(*RSS_i_*,*RSS_k_*) = 0. The similarity of one fingerprint to itself *s*(*RSS_k_*,*RSS_k_*) is set to *P_r_*. The four formulas below illuminate how RSS fingerprints generate support and suitability evaluation:

(11) $$r(RSS_{i},RSS_{k})=s(RSS_{i},RSS_{k})-\underset{k^{\prime }s.t.k^{\prime }\neq k}{\text{max}}\{a(RSS_{i},RSS_{k^{\prime }})+s(RSS_{i},RSS_{k^{\prime }})\}(i\neq k)$$

(12) $$r(RSS_{k},RSS_{k})=s(RSS_{k},RSS_{k})-\underset{k^{\prime }s.t.k^{\prime }\neq k}{\text{max}}\{a(RSS_{k},RSS_{k^{\prime }})+s(RSS_{k},RSS_{k^{\prime }})\}$$

(13) $$a(RSS_{i},RSS_{k})=\text{min}\{0,r(RSS_{k},RSS_{k})+\sum_{i^{\prime }s.t.i^{\prime }\notin \{i,k\}}\text{max}\{0,r(RSS_{i^{\prime }},RSS_{k})\}\}(i\neq k)$$

(14) $$a(RSS_{k},RSS_{k})=\sum_{i^{\prime }s.t.i^{\prime }\notin \{i,k\}}\text{max}\{0,r(RSS_{i^{\prime }},RSS_{k})\}$$

where both *RSS_k_*_’_ and *RSS_k_* are candidate fingerprints to compete to be the cluster representative, *RSS_i’_* is other fingerprints except *RSS_i_* and *RSS_k_*. After all fingerprints have been implemented the support evaluation and suitability evaluation by other fingerprints in the network, the cluster representatives and their subordinates can be identified through the following conditions:

(1) Fingerprint *RSS_k_* is chosen to be cluster representative when *r*(*RSS_k_*,*RSS_k_*) + *a*(*RSS_k_*,*RSS_k_*) > 0;
(2) Fingerprint *RSS_i_* is subordinate to *RSS_k_* when *RSS_k_* maximizes *r*(*RSS_i_*,*RSS_k_*) + *a*(*RSS_i_*,*RSS_k_*)(*i* ≠ *k*).

Every fingerprint makes the support evaluation of candidate representative taking into account the clustering similarity between itself and other RSS fingerprint representative candidates, along with the suitability evaluation of other RSS fingerprints as representatives. Every fingerprint will consider other fingerprints’ support evaluations to determine its suitability evaluation as a candidate fingerprint representative. Finally, if *r*(*RSS_k_*,*RSS_k_*) + *a*(*RSS_k_*,*RSS_k_*) > 0, fingerprint *RSS_k_* can be the representative. Those fingerprints that are not representatives would calculate the clustering similarities between themselves and representatives, and then decide the representative they belong to according to the maximum similarity principle. To restrict the overall computation cost, the evaluation range of a RSS fingerprint is limited to those RSS fingerprints that have at least 20% identical Aps, so by using the AP-Cluster method and similarities between RSS fingerprints, the crowdsourced fingerprints are clustered and RSS fingerprint representatives can be obtained efficiently, which facilitates the construction of a radio-map afterwards.

Note that the doors’ RSS fingerprints are included in the acquired clustering results. Since the doors of the indoor area can be considered as the anchors and their geographical locations are known, the fingerprint representatives of those fingerprints recorded in a small neighboring area of room door are adjusted to D-fingerprints. The other fingerprint representatives in clustering results which are unaware of their physical locations are referred to as C-fingerprints.

Rule 7: Given that *RSS_i_* (*i* = 1,2,…,*h*) are the representative fingerprints after clustering the crowdsourced fingerprints and some of the representative fingerprints *RSS_j_* (*j* ≤ *h*) are similar to the D-fingerprints (Rule 2). Except for the fingerprints *RSS_j_* (*j* ≤ *h*), the remaining representative fingerprints are the C-fingerprints.

### 4.4. Fingerprint Partition

**Figure 10:**
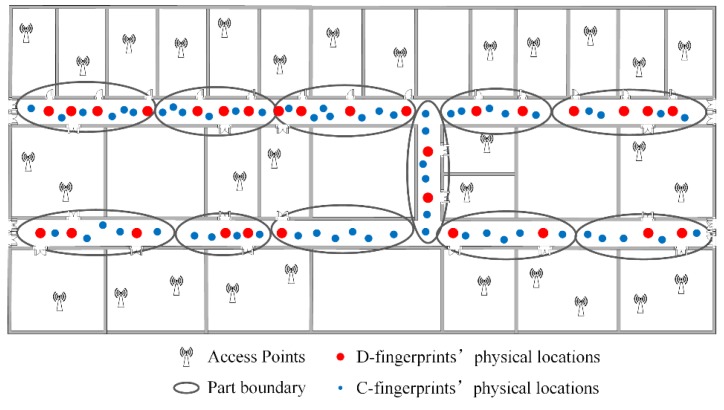
The schematic drawing of fingerprints partition.

To ensure the accuracy of the constructed radio-map, we narrow down the geographical scope to determine the physical locations of C-fingerprints, and all fingerprints including D-fingerprints and C-fingerprints are partitioned into a few parts. Since the physical locations of D-fingerprints are determined already, the corresponding geographical corridor areas including certain D-fingerprints can be confirmed as well. The RSS fingerprints are similar when their corresponding physical locations are in close proximity to each other. The fingerprints whose corresponding physical locations are close can be divided into the same part. The fingerprints partition would not care about the integrity of geographical corridor of every part. For example, a straight corridor could be partitioned into several small areas, or the public area of two vertical crossing corridors could be assigned to one area. K-means method [38] can classify the data into the specified number of parts. The number of parts can be set and employed to realize the fingerprints partition. Figure 10 gives the schematic drawing of fingerprints partition for all D-fingerprints and C-fingerprints. As seen from Figure 10, every part contains multiple D-fingerprints, and the physical locations of D-fingerprints determine the corresponding geographical corridor areas of the parts. The C-fingerprints in each part are not labeled with their physical locations.

### 4.5. Radio-Map Construction

**Figure 11:**
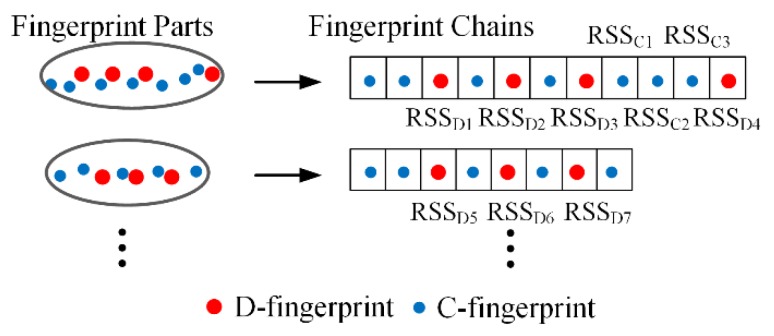
The generation of fingerprint chain.

According to the similarity between fingerprints, all the D-fingerprints and C-fingerprints in a fingerprint part can be arranged into an orderly fingerprint chain. The positions of C-fingerprints in fingerprint chain can be determined successively according to their similiarity degree to D-fingerprints in the fingerprint chain, as depicted in Figure 11. In Figure 11, there are three C-fingerprints *RSS*_*C*1_, *RSS*_*C*2_ and *RSS*_*C*3_ between D-fingerprints *RSS*_*D*3_ and *RSS*_*D*4_. The position of C-fingerprints *RSS*_*C*1_ and *RSS*_*C*3_ in the fingerprint chain can firstly be confirmed because among the C-fingerprints between *RSS*_*D*3_ and *RSS*_*D*4_, *RSS*_*C*1_ and *RSS*_*C*3_ are most similar to *RSS*_*D*3_ and *RSS*_*D*4_, respectively. Later, the position of C-fingerprint *RSS*_*C*3_ in the fingerprint chain can be decided. The sequential relationships between C-fingerprints in the generated fingerprint chain are the same as the sequential relationships between their corresponding physical locations.

The physical locations of C-fingerprints can be determined by using the physical locations of D-fingerprints, the physical distances between two adjacent doors as well as the similarities between C-fingerprints and D-fingerprints. The steps are as follows.

(1) Calculate the physical distance *d* between two doors whose D-fingerprints are both nearest to C-fingerprint based on the reference of fingerprint chain (e.g., the physical distance *d*_*D*3*D*4_ between door *D*_3_ and door *D*_4_ in Figure 11).
(2) Calculate the similarity δ between the two doors’ D-fingerprints (e.g., the similarity *δ*_*D*3*D*4_ between door *D*_3_’s D-fingerprint *RSS*_*D*3_ and door *D*_4_’s D-fingerprint *RSS*_*D*4_).
(3) Calculate the similarity δ’ between C-fingerprint and the D-fingerprint that is closest to C-fingerprint in fingerprint chain (e.g., the similarity δ_*C*1*D*3_ between *RSS*_*C*1_ and *RSS*_*D*3_).
(4) Calculate the physical distance Δ*d* between C-fingerprint and the D-fingerprint that is closest to the C-fingerprint in the fingerprint chain (e.g., the distance Δ*d*_*C*1*D*3_ between C-fingerprint *RSS*_*C*1_ and D-fingerprint *RSS*_*D*3_):
  (15) $$\Delta d=\frac{\delta^{\prime }}{\delta }\times d$$

According to the C-fingerprints’ sequence in fingerprints chain and the physical distance Δ*d*, the location of C-fingerprints is finally obtained.

**Figure 12:**
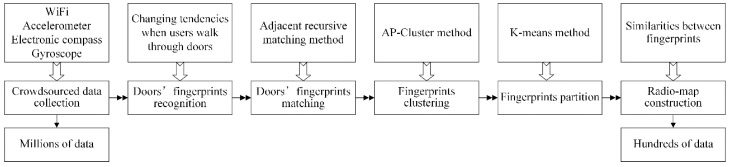
The procedure of radio-map automatic construction.

To sum up, the doors’ D-fingerprints are identified from the crowdsourced fingerprints including RSS fingerprints, walking acceleration, changing tendencies of walking direction and rotational angular velocity when users walk through doors firstly. The doors’ D-fingerprints are matched according to the feature that the least time and minimum steps passing through two adjacent doors in a single user’s trajectory in the second step. Thirdly, the crowdsourced fingerprints are clustered to representative fingerprints based on the AP-Cluster method. Then, the fingerprints are partitioned based on the K-means method in the fourth step. Finally, the radio-map is constructed on the basis of the similarity of fingerprints. The whole process is summarized as Figure 12. The RACC algorithm performs the characteristic mining and matching to millions of crowdsourced data, and extracts hundreds of representative RSS fingerprints labeled with physical locations, and provides a reference to online localization.

### 4.6. An Example of RACC

**Figure 13:**
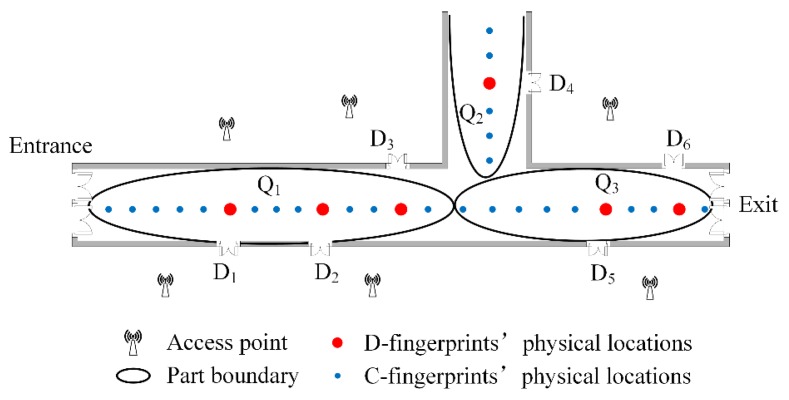
A simple example of RACC algorithm.

The main idea of RACC is to find the doors’ fingerprints in crowdsourced fingerprints firstly (based on the changing tendencies of users’ smartphone sensors) and their corresponding physical locations. Once the doors’ locations are confirmed, the anchors of the whole radio-map are fixed. Then, according to the D-fingerprints and their locations, the representative fingerprints (C-fingerprints) clustered from the crowdsourced fingerprints are placed between D-fingerprints based on the similarity. The whole radio-map is constructed. Here is an example of RACC, as shown in Figure 13.

The steps of RACC is briefly described below.

(1) D-fingerprints are recognized. Users’ walking direction and rotational angular velocity will change when users traverse doors *D*_1_~*D*_6_. Therefore, doors *D*_1_~*D*_6_ are identified and the fingerprints (D-fingerprints) near them are recorded, as the red dots show in Figure 13.
(2) D-fingerprints are matched to their physical locations. According to the geographical constraints between doors *D*_1_~*D*_6_ and users’ walking trajectories, *D*_1_~*D*_6_’s fingerprints can be matched to their corresponding physical locations.
(3) C-fingerprints are selected. The crowdsourced fingerprints collected in the area are clustered using the AP-Cluster method to obtain the representative fingerprints (C-fingerprints), as the blue dots shown in Figure 13.
(4) Fingerprints are partitioned. All fingerprints (D-fingerprints and C-fingerprints) in the area are partitioned to three parts: *Q*_1_, *Q*_2_ and *Q*_3_.
(5) The radio-map is constructed. For every part, C-fingerprints’ physical locations can be calculated based on the similarity between D-fingerprints and C-fingerprints. When all C-fingerprints are linked to their corresponding physical locations, the radio map is constructed.

## 5. Performance Evaluation

**Figure 14:**
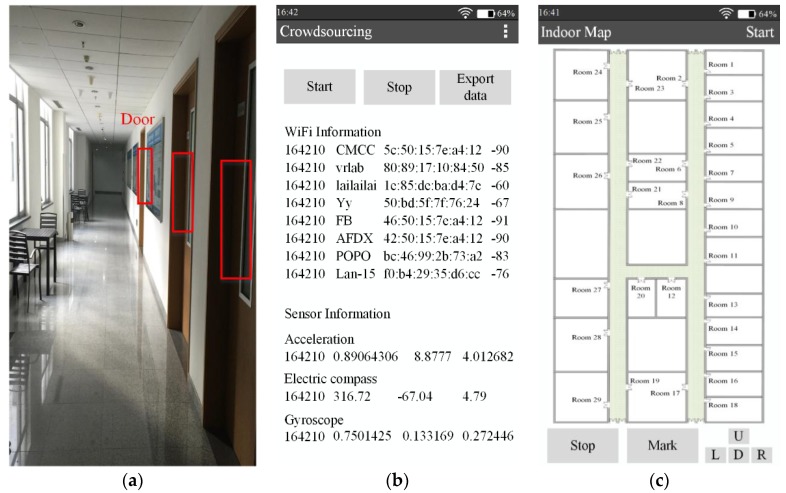
Preparation for experiment. (**a**) A corridor in the experimental area; (**b**) The crowdsourced data collection interface; (**c**) The interface of test points’ fingerprints collection.

In this section, the experiments are conducted to study the performance of RACC on one floor of a typical office building covering 3600 m^2^, in which corridors cover 385 m^2^ and the total number of room doors is 29, as illustrated in Figure 2 and Figure 14a. Figure 14a shows a certain corridor in the experimental area, which contains three doors (marked by red boxes).

A number of APs are randomly deployed in the experiment area and these APs’ physical locations are unknown. Since some deviations would occur in the RSS fingerprints and sensor measurements at the same location from using different smartphones, the crowdsourced data of RACC are all collected on the same type of smartphone (Xiaomi, Android OS 4.2) which supports WiFi and is fully equipped with sensors. A software app is designed and installed on the smartphone to collect and record WiFi information of locations that users walk past, users’ walking direction and rotational angular velocity, as shown in Figure 14b. The sampling frequency is 1 Hz. Ten volunteers took part in the data collection. They all carry a smartphone and walk in the experimental area while performing their daily activities, in the meantime, each smartphone carries out its data collection for a total five workdays (from Monday to Friday) during the period of 8:00~18:00. The experiment configuration is shown in Table 2.

In totally 194 APs were scanned and 546 effective user traces were collected. These traces cover most corridor areas and several room areas. To validate the effectiveness of the automatically constructed radio-map, locations in corridors are selected as the test points for online localization. At every test point, the received APs’ MAC addresses, their corresponding RSS value as well as actual locations are recorded. Figure 14c shows the software interface that can manually mark the test points’ physical locations.

### 5.1. Smartphone Measurement Analysis

Since the change tendencies of the electronic compass and gyroscope in users’ smartphones are used to realize the D-fingerprints’ recognition, the measurement sensitivity of users’ smartphone sensors was analyzed.

**Figure 15:**
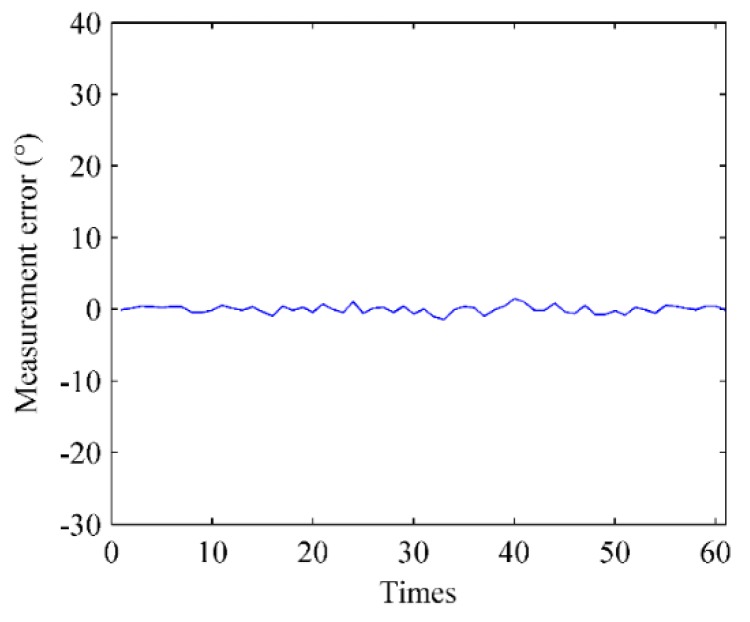
The measurement sensitivity of the electronic compass in smartphones.

The measurement sensitivity of the electronic compass was evaluated first. Figure 15 shows the measurement error of the electronic compass when smartphones rotate 90° manually. For 60 measurements, the errors range between −2°~+2° (the actual measurements are 90 ± 2°), which has little effect on the fingerprint recognition.

**Figure 16:**
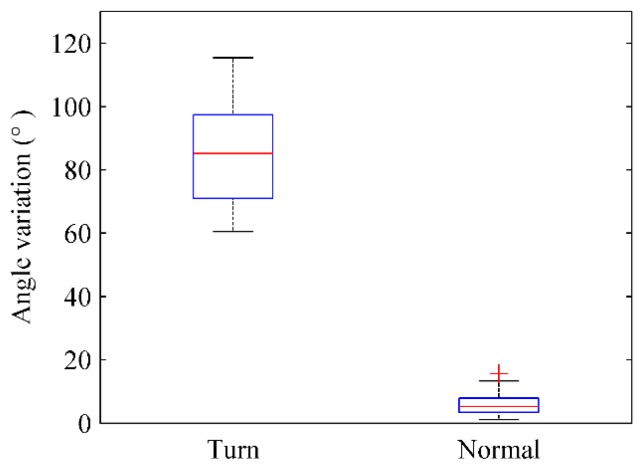
The angular variation of the electronic compass (making a turn *vs.* walking normally in corridors).

Figure 16 depicts the angular variation of the electronic compass when users traverse through doors or walk normally in corridors. When users make a turn to walk in or out of doors, the angular variation range of the electronic compass is 60°~115° and its median value is approximately 90°. When users walk normally in corridors, the angular variation of electronic compass is less than 15° and the median value is approximately 5°. Therefore, users’ turns at doors can be clearly identified by the angular variation of the electronic compass.

**Figure 17:**
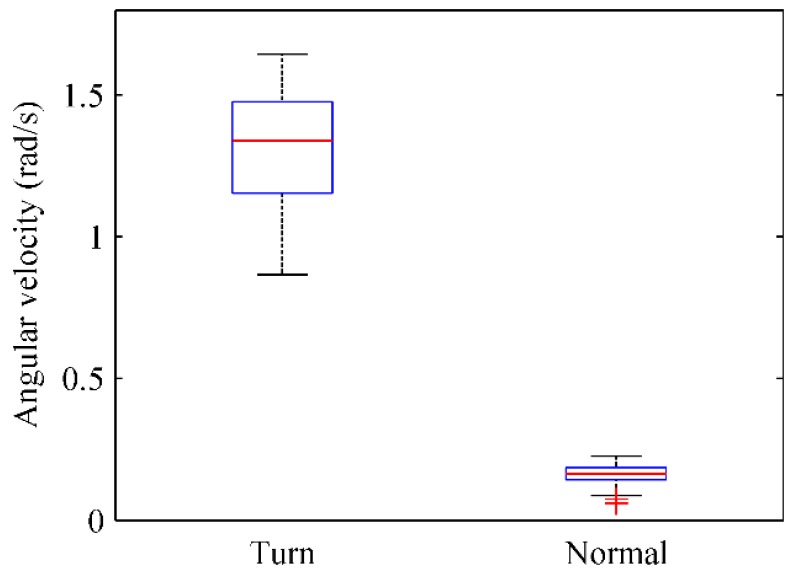
The angular velocity variation of the gyroscope (making a turn *vs.* walking normally in corridors).

Figure 17 illustrates the angular velocity variation of the gyroscope when users traverse through doors or walk normally in corridors. When users make a turn to walk in or out doors, the angular velocity variation of the gyroscope fluctuates from 1.2 rad/s to 1.5 rad/s and its median value is approximately 1.3 rad/s. When users walk normally in corridors, the angular velocity fluctuation of gyroscope is less than 0.3 rad/s and the median value is approximately 0.2 rad/s. Thus, users’ turning at doors can also be clearly identified by the angular velocity variation of the gyroscope.

Therefore, if changes happen in both the electronic compass and gyroscope, users’ turning moments can be effectively determined. The predefined thresholds of *ε_cp_* and *ε_rot_* in Rule 1 could be set as 70° and 1.2 rad/s, respectively, as well in this case.

**Figure 18:**
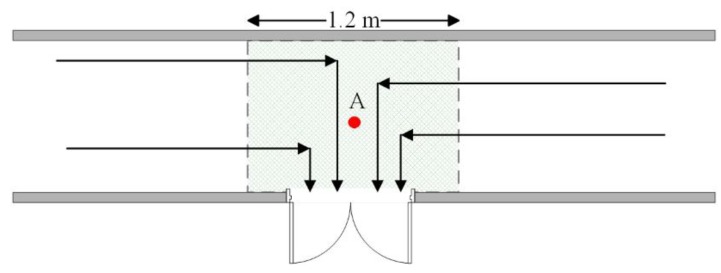
The fingerprint sampling in front of a door.

**Figure 19:**
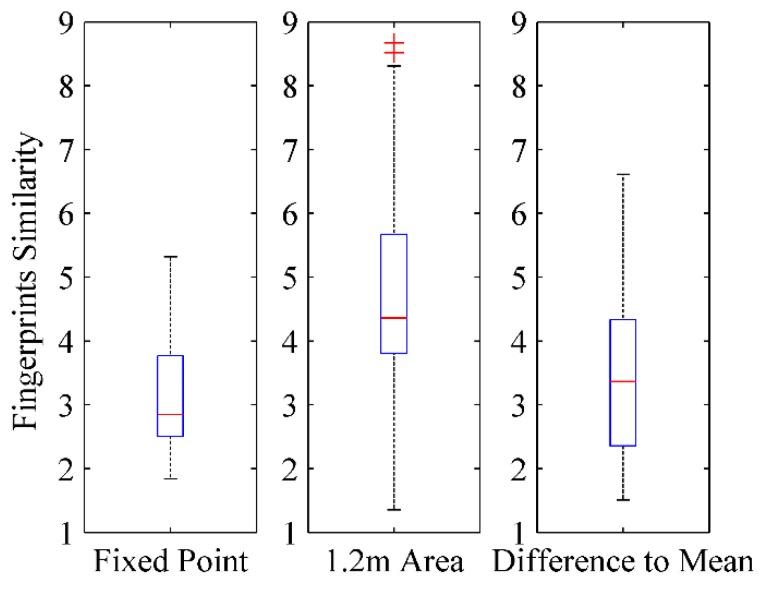
The analysis of RSS fingerprint measurements.

The fluctuation of RSS fingerprint measurements is studied too. As shown in Figure 18, 500 fingerprints within a 1.2 m area in front of a door are sampled. The collected fingerprints almost cover the shadow area. In addition, a fixed point measurement at point A in front of the door is executed, and data from 120 fingerprints are acquired. In total 16 APs are scanned and the RSS values of the APs range between −60 dBm~−95 dBm. The analysis of RSS fingerprint measurements is given in Figure 19. For the 120 RSS fingerprints measured at the same fixed point, the RSS similarity (Rule 2) is mainly distributed in the range of 2.5~3.8 dB and the maximum similarity difference is less than 5.3. For the 500 fingerprints sampled within a 1.2 m area in front of the door, the similarity between any two fingerprints mainly fluctuates within the range of 3.8~5.7 and the maximum fingerprint similarity difference is less than 9. The average value of 500 fingerprints is also calculated as D-fingerprints (Rule 3). The similarity between any of the 500 fingerprints and the average value mostly ranges between 2.3~4.3 and the maximum similarity difference is less than 6.6. So when users make a turn to walk in or out of the door, the similarity between any collected fingerprint in front of the door is less than 9. The RSS fingerprint dissimilarity threshold *ε_RSS_* (Rule 2) can be set to 9 in this case and the fingerprints sampled within 1.2 m area in front of the door can be merged into the D-fingerprint.

### 5.2. Results of Doors’ Fingerprint Recognition and Matching

When the walking direction and rotational angular velocity in crowdsourced sensor measurements change dramatically, the number of user trajectories passing through the corresponding RSS fingerprints are analyzed statistically according to Rule 1 and 2 mentioned in Section 4.1. Table 3 gives the results in descending order.

**Figure 20:**
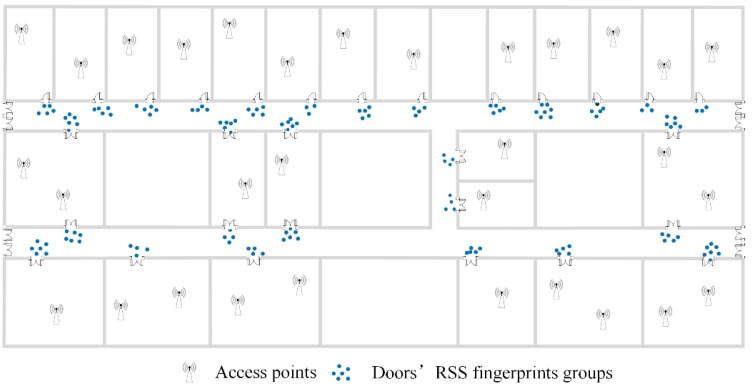
The recognition of doors’ fingerprint groups.

**Figure 21:**
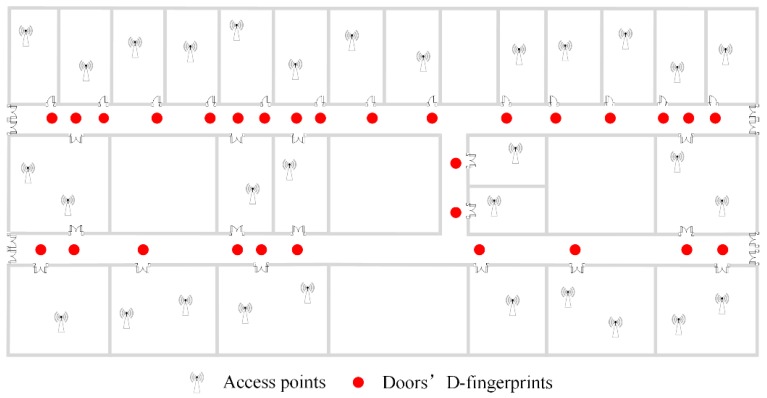
Doors’ D-fingerprints.

**Figure 22:**
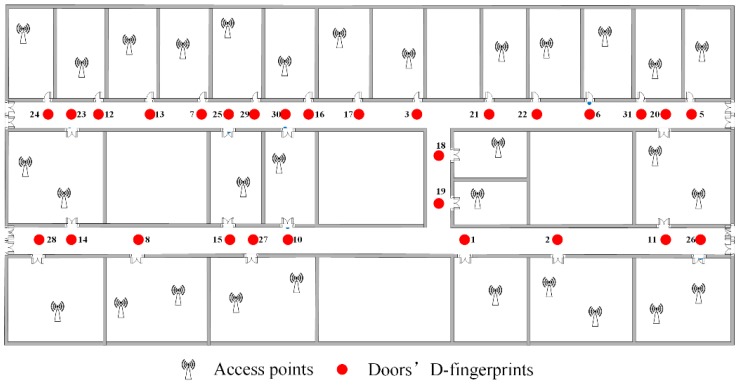
The matching of D-fingerprint.

From Table 3, there are 40 groups of RSS fingerprints that meet the requirements of Rules 1 and 2. The more the number of user trajectories that pass through a certain RSS fingerprint group is, the higher the possibility of this RSS fingerprint group corresponds to a room door because of the statistical characteristics of our crowdsourced data. The top 31 RSS fingerprint groups are determined to correspond to room doors and corridor intersections. The remaining RSS fingerprint groups are then regarded as the outliers through which users walk abnormally. The No. 4 RSS fingerprint group and No. 9 RSS fingerprint group are recognized to correspond to corridor intersections based on Rule 4 in Section 4.1. Figure 20 shows the recognition results of doors’ fingerprint groups and Figure 21 shows the doors’ D-fingerprints. It should be noted that Figure 20 and Figure 21 merely illustrate the extraction of doors’ fingerprint groups or D-fingerprints from crowdsourced fingerprints. Each fingerprint group or D-fingerprint in Figure 20 or Figure 21 are not annotated to a specific room door. Figure 22 displays the matching results of D-fingerprints.

As seen from Figure 20, Figure 21 and Figure 22, doors’ RSS fingerprints can be efficiently identified and labeled following the recognition and matching procedures in RACC. Assigning physical locations to D-fingerprints provides references for the computation of other RSS fingerprints’ physical positions.

### 5.3. Results of Radio-map Construction

From Section 4.3, the input preference *P_r_* would influence the number of RSS fingerprint clusters:

(16) $$P_{r}=\gamma \cdot \frac{\sum_{i=1}^{n}\sum_{k=1}^{n}s(RSS_{i},RSS_{k})}{n}(i\neq k)$$

where γ is the preference coefficient, *n* is the number of crowdsourced fingerprints, *s*(*RSS_i_*,*RSS_k_*) is the clustering similarity between *RSS_i_* and *RSS_k_*.

**Figure 23:**
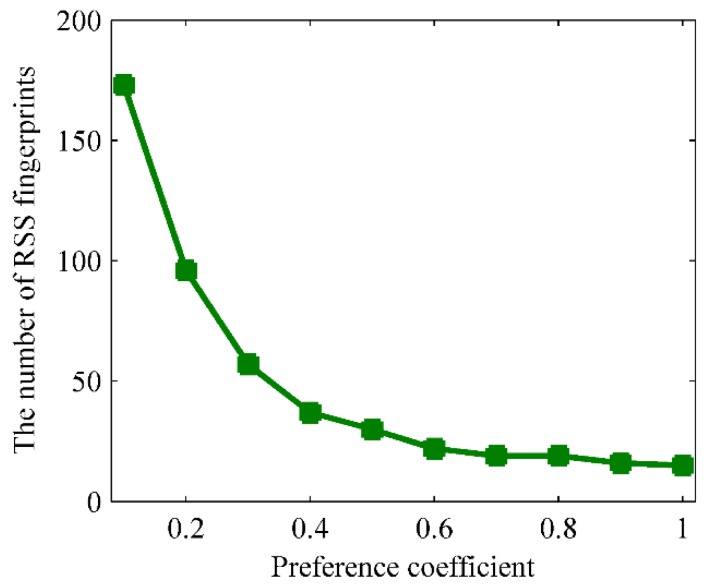
The number of fingerprint clusters *versus* preference coefficient.

Figure 23 investigates the relationship between the preference coefficient and the number of fingerprint clusters when 0 < γ ≤ 1. It can be concluded from Figure 23 that the number of fingerprint clusters would reduce gradually with the increasing preference coefficient. Since the number of fingerprint clusters directly determines the size of the radio-map, the bigger the radio-map is, the more reference information it provides for online localization.

**Figure 24:**
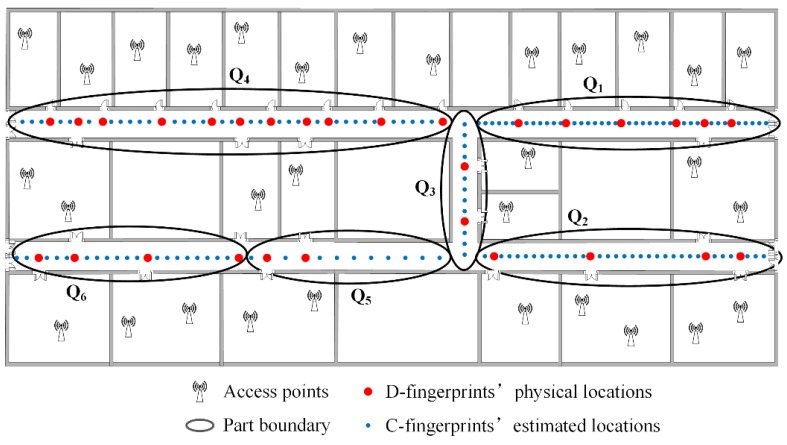
The automatic constructed radio map when γ = 0.1, *k* = 6.

**Figure 25:**
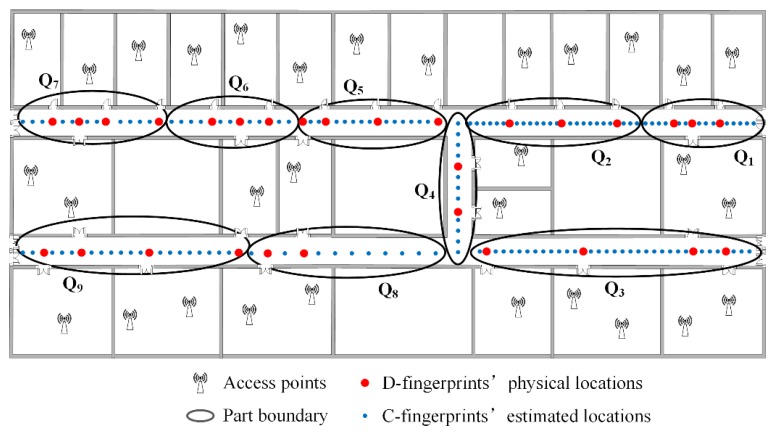
The automatic constructed radio map when γ = 0.1, *k* = 9.

**Figure 26:**
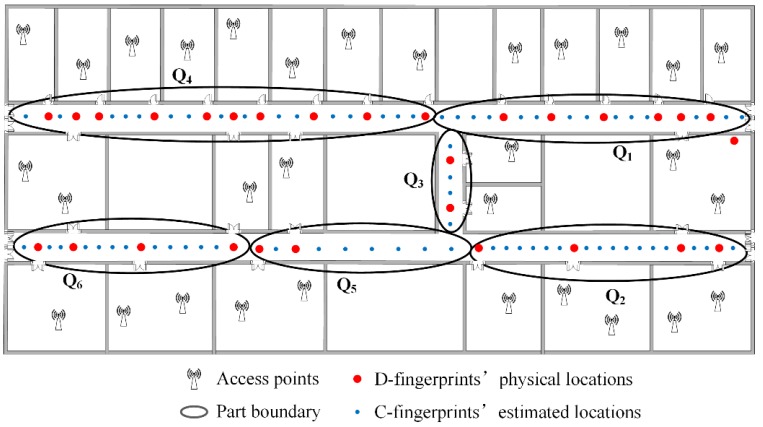
The automatic constructed radio map when γ = 0.2, *k* = 6.

**Figure 27:**
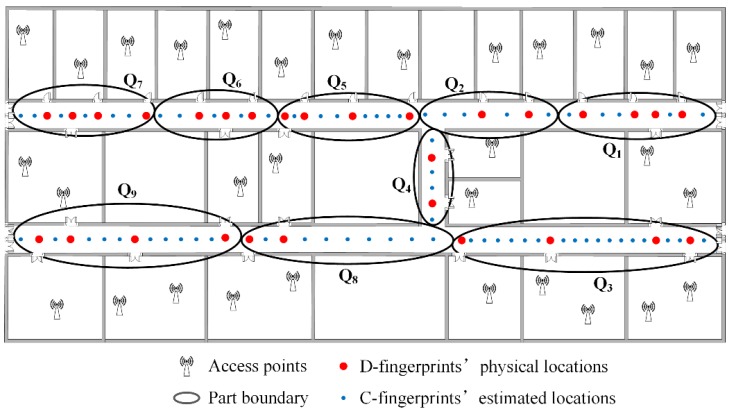
The automatically constructed radio-map when γ = 0.2, *k* = 9.

The fingerprints partition needs to set the number of parts in advance and every part must contain at least two doors’ D-fingerprints. The locations of C-fingerprints in every part can be estimated by referencing the locations of D-fingerprints according to the steps in Section 4.5. The total number of doors in the experimental area is 29, and each part has to contain at least 2 doors, thus the maximum number of parts is 29/2 = 14.5. The parts number *k* can be set to the integer between 1~14. On this basis, we pick two typical cases *k* = 6 and *k* = 9 as the number of parts. In accordance with the clustering, partitioning and calculating procedures of RACC, the radio-map construction results when γ = 0.1, *k* = 6; γ = 0.1, *k* = 9; γ = 0.2, *k* = 6 and γ = 0.2, *k* = 9 are given in Figure 24, Figure 25, Figure 26 and Figure 27, respectively.

From Figure 24, Figure 25, Figure 26 and Figure 27, it can be observed that the crowdsourced RSS fingerprints are clustered and partitioned by utilizing the AP-Cluster method and K-means method. The RSS fingerprints in small neighboring areas can be gathered into a cluster and the selected RSS fingerprint representatives can reflect the fingerprint features of their subordinates.

### 5.4. Localization Results 

#### 5.4.1. Localization Based on Parts of Radio-Map

**Figure 28:**
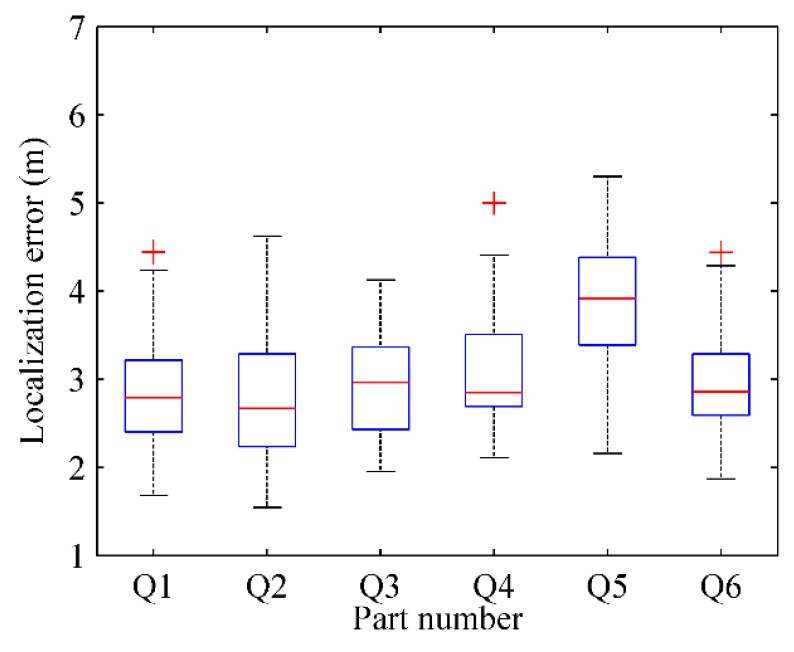
The localization results of parts in radio map when γ = 0.1, *k* = 6.

**Figure 29:**
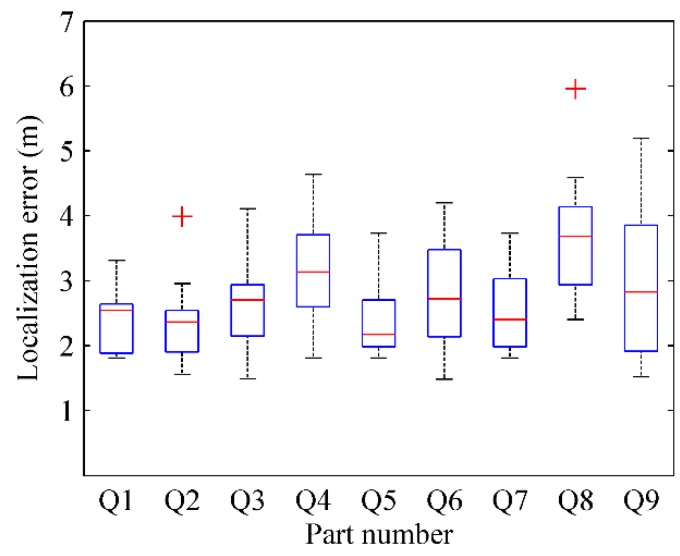
The localization results of parts in radio map when γ = 0.1, *k* = 9.

**Figure 30:**
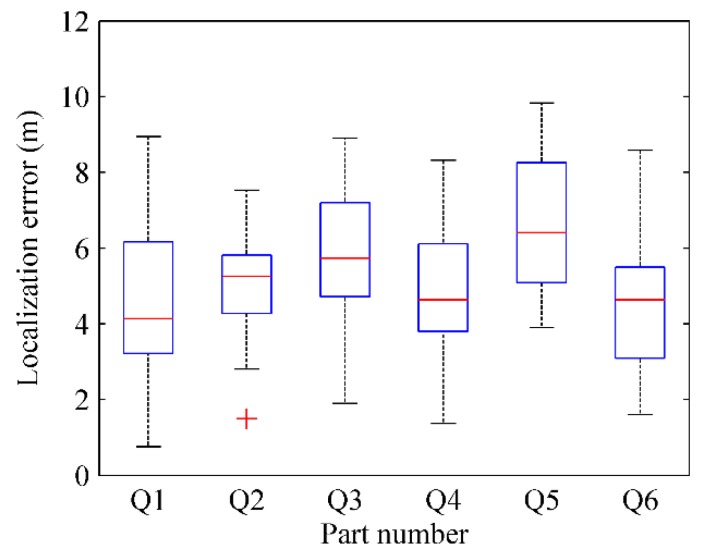
The localization results of parts in radio map when γ = 0.2, *k* = 6.

**Figure 31:**
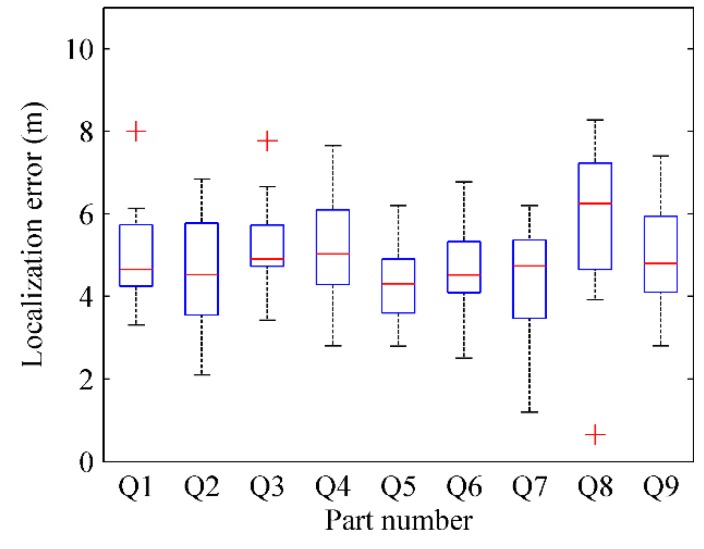
The localization results of parts in radio map when γ = 0.2, *k* = 9.

To validate the performance of RACC, the online localization is carried out based on the weighted k-nearest neighboring method at 110 test points in the experiment area. The calculation of localization error is:

(17) $$LE=\sqrt{{\left(x_{k}^{e}-x_{k}^{r}\right)}^{2}+{\left(y_{k}^{e}-y_{k}^{r}\right)}^{2}}$$

where $P_{k}^{r}(x_{k}^{r},y_{k}^{r})$ is the actual physical location of test point *k* and $P_{k}^{e}(x_{k}^{e},y_{k}^{e})$ is the estimated physical location by RACC. The localization result of every part in four radio maps are respectively depicted in Figure 28, Figure 29, Figure 30 and Figure 31.

From Figure 28 and Figure 29, when γ = 0.1, *k* = 6, the localization error of each part in the radio-map is mostly in the range of 2.5 m~4.5 m, and especially the localization error of the Q5 part is larger than that of other parts. When γ = 0.1, *k* = 9, the localization error of each part in the radio-map ranges roughly from 2.5 m to 4 m, and the localization error of Q8 part in particular is larger than that of other parts. The reason is that the Q5 part and Q8 part correspond to the same area in the physical environment in which only two room doors are located, so less location information is available for reference. Furthermore, APs are sparsely distributed in this area resulting in a tiny difference between RSS fingerprints. Accordingly, since the fingerprint chains are generated with relative deviation among fingerprints, the accuracy of radio map decreases, which leads to an increased localization error.

From Figure 30 and Figure 31, when γ = 0.2, *k* = 6, the localization error of each part in the automatically constructed radio-map is nearly from 4 m to 7 m, while it is almost in the scope of 4 m~6 m when γ = 0.2, *k* = 9. This indicates that properly increasing the number of parts would improve the accuracy of the generated radio-map if each part contains at least two room doors’ D-fingerprints. The reason is that each part will have less fingerprints when the number of parts increases, thus more uncertain aspects are removed when the fingerprint chain is ordered, and a more accurate RSS fingerprint chain can be obtained accordingly.

One thing that needs to be pointed out is that the localization error of RACC from 2.5 m to 4 m (γ = 0.1, *k* = 9) or from 4 m to 6 m (γ = 0.2, *k* = 9) is not a very accurate localization. For some parts of the constructed radio-map, the accuracy is no better than that of the manually constructed ones. However, considering the combination of expenditure in fingerprint collection and benefit in localization accuracy, RACC is more practical than traditional data sampling methods. RACC can construct the radio-map of indoor areas automatically without participation of sampling technicians, which dramatically reduces the cost of fingerprint database construction. This is of great significance for the application and promotion of indoor fingerprint-based localization techniques.

From the comparison between Figure 28 and Figure 30 (or Figure 29 and Figure 31), it can also be learned that the average localization performance when γ = 0.1 is better than that when γ = 0.2, which means the setting of preference coefficient in fingerprint clustering has a great impact on the localization accuracy. The smaller the preference coefficient is, the more RSS fingerprints a radio-map contains, and the more accurately users are located. The reason is that the radio-map serves as a database which offers online searching and matching, so user’s current fingerprints can be matched to a higher degree if the radio-map contains more fingerprints. However, the size of the radio-map should not be too large, otherwise it would occupy too much storage space and take more matching time, which results in online localization latency.

#### 5.4.2. Impact of Parameters on Localization

**Figure 32:**
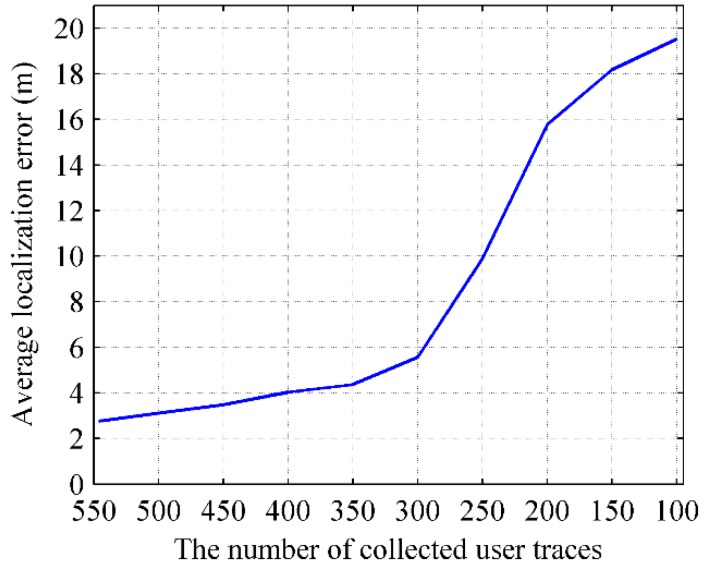
Relationship between the number of users’ traces and localization error.

The relationship between the localization error and number of users’ walking trajectories is shown in Figure 32. The localization precision of RACC will reduce with the decrease of the number of users’ walking trajectories. The averaged localization error is no more than 5.8 m when the number of users’ walking trajectories is no less than 300, whereas the averaged localization error is about 16 m when the number of users’ walking trajectories is only 200. It is because that RACC is a localization method based on crowdsourced data, it needs a lot of ergodic crowdsourced data to construct a radio- map automatically. Therefore, when the number of users’ walking trajectories reduces to a threshold, the ergodicity of crowdsourced data will be weakened correspondingly. For instance, if there are some doors where users seldom or never walk by, major deviations will occur during the matching process of these doors in the RACC algorithm, which will also cause an increase in the average localization error. Thus it is necessary to ensure the ergodicity of crowdsourced data in order to guarantee the effectiveness of RACC.

**Figure 33:**
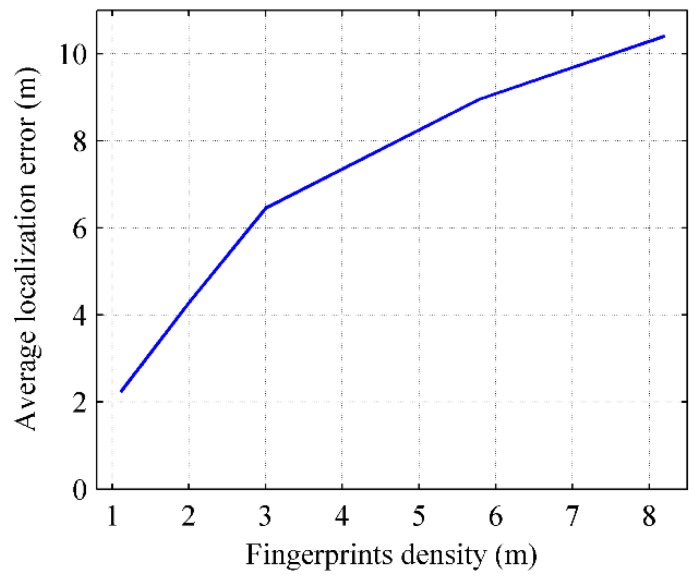
Relationship between the fingerprint density and localization error.

When the preference coefficient γ is picked as 0.1, 0.2, 0.3, 0.4 and 0.5, the number of fingerprint clusters are 171, 96, 57, 37 and 30, respectively, and the corresponding density of the sampling position of fingerprints are 1.1 m, 2 m, 3 m, 5.7 m and 8.3 m apart. The relationship between the localization error and the fingerprint density is shown in Figure 33.

The localization error increases with the reduction of the fingerprint density. The reason is that effective reference information is reduced when the number of the representative fingerprints decreases, and then the localization precision is influenced.

RACC uses the AP-Cluster method to cluster the crowdsourced fingerprints. By controlling the preference coefficient γ in the AP-Cluster method, a suitable radio-map fingerprint density can be obtained and thus the localization accuracy is improved. Since the number of clustered RSS fingerprints determines the size of the radio-map, the more fingerprints the radio-map has, and the more reference information it provides, the higher the localization accuracy will be. On the other hand, the matching calculation in online localization will increase simultaneously.

#### 5.4.3. Comparison with Manual Calibration

The localization performance of RACC is compared with that of a manual fingerprints calibration. In corridors of the experimental area, sampling points are set every 2 m, and 60 fingerprints are collected at each point. The average value of 60 fingerprints is considered as the sampling points’ RSS fingerprint. A total of 96 points are sampled and the manually calibrated radio- map is generated containing 96 RSS fingerprints. The radio-map constructed by RACC when γ = 0.2, *k* = 9 also has 96 fingerprints and the average distance between fingerprints is 2 m. Detailed information is shown in Table 4.

**Figure 34:**
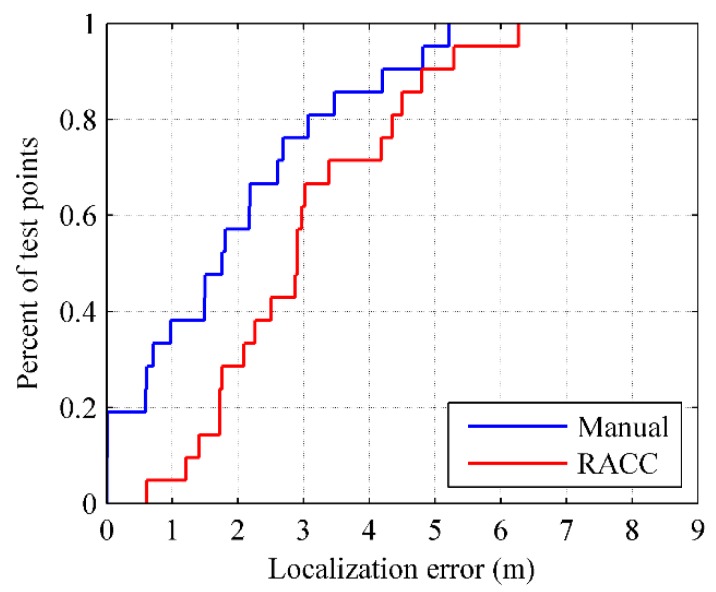
Localization results of manual calibration and RACC.

For the two radio-maps constructed by manual calibration and RACC, we utilize the test points to perform the online localization based on the weighted kNN method (Section 3.3). The localization results are shown in Figure 34.

The 60%ile and 80%ile localization errors of RACC are respectively 3 m and 4.3 m, while the 60%ile and 80%ile localization errors of manual calibration are respectively 2.2 m and 3.1 m, which means the localization accuracy of RACC can reach about 70% of the localization accuracy of manual calibration. However, as an automatic construction method, RACC reduces greatly the cost of fingerprint sampling and radio-map construction, effectively solving the resource-consumption problem when a location fingerprint database is established.

#### 5.4.4. Comparison with Other Localization Methods

The localization performance of RACC are compared with that of Zee [14] and LiFS [35], as shown in Table 5. We can see from the Table 5 that RACC takes advantage of users’ smartphone sensors (accelerometer, gyroscope and compass) and produces comparable localization accuracy for indoor corridors.

The key feature of RACC is that it uses the changing tendencies of users’ smartphone sensors to identify the indoor anchors (doors) and regards the locations of doors as the fixed reference of the whole radio-map. The representative fingerprints from the crowdsourced data can be obtained by the AP-Cluster method and the radio-map is constructed after linking the representative fingerprints to their corresponding physical locations on the basis of the fixed reference and the similarity between fingerprints.

The complex environment in indoor buildings can provide a lot of potential available anchor information. The GPS signal strength would greatly decrease when users enter the building, which will help to determine that users are at a certain building entrance. The accelerometer in users’ smartphones would fluctuate considerably when the elevator starts or stops, which will help determine that the current location is a certain elevator’s location. At some places in corridors, there might exists a distinctive turning angle, or a four-star strength GPS signal can be received, or no WiFi or GSM signal can be detected. Those characteristics can be used as possible anchors for radio-map construction. Increasing the number of anchors will provide more reference information, reduce the radio-map construction errors and thus improve the localization accuracy.

All in all, the proposed algorithm RACC utilizes crowdsourced WiFi information and sensor measurements to construct the radio map automatically. It greatly reduces the resource consumption of offline data collection while ensuring an acceptable localization accuracy, and thus has the merits of flexibility and extensibility over traditional methods.

## 6. Conclusions

In this paper, an automatic radio-map construction algorithm based on crowdsourcing is proposed to solve the problem of high cost and poor extensibility of offline fingerprint collection in traditional fingerprint-based localization algorithms. Based on the proposed RACC algorithm, the crowdsourced WiFi information and sensor measurements can be implicitly collected through the smartphones carried by users, and doors’ RSS fingerprints can be identified according to their statistical features when users walk through doors. Doors’ fingerprints can be annotated with their corresponding physical locations according to the proposed adjacent recursive matching method. After fingerprints are clustered and partitioned based on AP-Cluster method and K-means method, the radio-map is finally constructed in accordance with the similarity. The experimental results show that RACC can efficiently solve the resource-consuming problem and achieve a competitive localization accuracy.

## Figures and Tables

**Table 1:** The recording format of crowdsourced data.

Croudsourced Data						
User ID		ID				
	Data Type	RSS Fingerprints		Acceleration	Walking Direction	Rotational Angular Velocity
Time		AP	RSS			
*t*_1_		${\left\{ap_{1},ap_{2},\ldots \right\}}_{t_{1}}$	$\left\{rss_{11},rss_{12},\ldots \right\}$	$acc_{t_{1}}$	$cp_{t_{1}}$	$rot_{t_{1}}$
*t*_2_		${\left\{ap_{1},ap_{2},\ldots \right\}}_{t_{2}}$	$\left\{rss_{21},rss_{22},\ldots \right\}$	$acc_{t_{2}}$	$cp_{t_{2}}$	$rot_{t_{2}}$
…		…	…	…	…	…
*t*_s_		${\left\{ap_{1},ap_{2},\ldots \right\}}_{t_{s}}$	$\left\{rss_{s1},rss_{s2},\ldots \right\}$	$acc_{t_{s}}$	$cp_{t_{s}}$	$rot_{t_{s}}$

**Table 2:** The experiment configuration of RACC.

Floor Area (m^2^)	3600	Sampling Frequency	1 Hz
Corridor area (m^2^)	385	Sampling time	8:00~18:00
Number of volunteers	10		

**Table 3:** Statistical analysis of the number of user traces when walking through the changing points.

Fingerprint Groups Number	Number of User Traces	Fingerprint Groups Number	Number of User Traces	Fingerprint Groups Number	Number of User Traces	Fingerprint Groups Number	Number of User Traces
**1**	54	**11**	38	**21**	24	**31**	16
**2**	52	**12**	36	**22**	24	**32**	6
**3**	52	**13**	32	**23**	22	**33**	5
**4**	50	**14**	28	**24**	22	**34**	3
**5**	50	**15**	28	**25**	22	**35**	2
**6**	46	**16**	28	**26**	20	**36**	2
**7**	44	**17**	26	**27**	18	**37**	1
**8**	44	**18**	26	**28**	18	**38**	1
**9**	42	**19**	26	**29**	18	**39**	1
**10**	40	**20**	26	**30**	18	**40**	1

**Table 4:** The information of radio map constructed by RACC (γ = 0.2, *k* = 9).

Part Number	Part Length (m)	Number of Doors	Number of RSS Fingerprints after Clustering	Average Distance between Fingerprints (m)
Q1	20	4	9	2.22
Q2	16	2	7	2.29
Q3	39	4	21	1.86
Q4	15	2	6	2.50
Q5	20	4	11	1.82
Q6	15	3	8	1.88
Q7	19	4	10	1.90
Q8	25	2	10	2.50
Q9	26	4	14	1.86
SUM	195	29	96	2.03

**Table 5:** Comparison with other localization methods.

Method	Sensors	Floor Plan	Experimental Areas	Reported 50%ile Accuracy	Reported 80%ile Accuracy	Fingerprint Density
Zee	Accelerometer	Yes	Corridor	7 m (ZeeHorus)	13 m (ZeeHorus)	-
				6 m (ZeeEZ)	13 m (ZeeEZ)	
				1.2 m (ZeeHorus-Path Only)	3 m (ZeeHorus-Path Only)	
	Gyroscope					
				1.0 m (ZeeEZ-Path Only)	2.3 m (ZeeEZ-Path Only)	
	Compass					
LIFS	Accelerometer	Yes	Corridor & Room	4.5 m (LIFS)	7 m (LIFS)	/4 m^2^
				1.5 m (LIFS-TM)	3.5 m (LIFS-TM)	
RACC	Accelerometer	Yes	Corridor	1.7 m	2.2 m	/1 m
	Gyroscope					
	Compass					
				2.9 m	4.3 m	/2 m
